# SMC3 contributes to heart development by regulating super-enhancer associated genes

**DOI:** 10.1038/s12276-024-01293-0

**Published:** 2024-08-01

**Authors:** Bowen Zhang, Yongchang Zhu, Zhen Zhang, Feizhen Wu, Xiaojing Ma, Wei Sheng, Ranran Dai, Zhenglong Guo, Weili Yan, Lili Hao, Guoying Huang, Duan Ma, Bingtao Hao, Jing Ma

**Affiliations:** 1 Key Laboratory of Metabolism and Molecular Medicine, Ministry of Education, Department of Biochemistry and Molecular Biology, School of Basic Medical Sciences; ENT Institute, Department of Facial Plastic and Reconstructive Surgery, Eye & ENT Hospital; Institute of Medical Genetics & Genomics; Key Laboratory of Birth Defects, Children’s Hospital; Medical Science Data Center at Intelligent Medicine Institute, Fudan University, Shanghai, 200032 China; 2 Henan Medical Genetics Institute, Henan Provincial Key Laboratory of Genetic Diseases and Functional Genomics, People’s Hospital of Zhengzhou University, Zhengzhou University, Zhengzhou, 450000 China; 3 Shanghai Pediatric Congenital Heart Disease Institute and Pediatric Translational Medicine Institute, Shanghai Children’s Medical Center, Shanghai Jiao Tong University School of Medicine, Shanghai, 200127 China; 4 Zhongshan School of Medicine, Sun Yat-Sen University, Guangzhou, Guangdong 510080 China; 5 Department of Immunology, School of Basic Medical Sciences, Zhengzhou University, Zhengzhou, Henan 450001 China; 6 Henan Eye Institute, Henan Academy of Innovations in Medical Science, Zhengzhou, Henan 450000 China

**Keywords:** Gene expression, Development, Congenital heart defects, Embryology

## Abstract

Abnormal cardiac development has been observed in individuals with Cornelia de Lange syndrome (CdLS) due to mutations in genes encoding members of the cohesin complex. However, the precise role of cohesin in heart development remains elusive. In this study, we aimed to elucidate the indispensable role of SMC3, a component of the cohesin complex, in cardiac development and its underlying mechanism. Our investigation revealed that CdLS patients with *SMC3* mutations have high rates of congenital heart disease (CHD). We utilized heart-specific *Smc3*-knockout (SMC3-cKO) mice, which exhibit varying degrees of outflow tract (OFT) abnormalities, to further explore this relationship. Additionally, we identified 16 rare *SMC3* variants with potential pathogenicity in individuals with isolated CHD. By employing single-nucleus RNA sequencing and chromosome conformation capture high-throughput genome-wide translocation sequencing, we revealed that *Smc3* deletion downregulates the expression of key genes, including *Ets2*, in OFT cardiac muscle cells by specifically decreasing interactions between super-enhancers (SEs) and promoters. Notably, Ets2-SE-null mice also exhibit delayed OFT development in the heart. Our research revealed a novel role for SMC3 in heart development via the regulation of SE-associated genes, suggesting its potential relevance as a CHD-related gene and providing crucial insights into the molecular basis of cardiac development.

## Cohesin Complex: SMC3’s Crucial Role in Congenital Heart Disease

Understanding heart development is vital as defects in this process are a major cause of birth abnormalities. This study focuses on a protein, SMC3, and its role in heart development. Experiments were conducted on mice genetically altered to lack SMC3 in heart cells. Researchers found that mice without SMC3 had various heart defects, like those seen in humans with congenital heart disease. They also found mutations in the SMC3 gene in patients with congenital heart disease, suggesting a link between SMC3 and heart development in humans. The findings reveal that SMC3 plays a crucial role in heart development, with its absence leading to significant heart defects in mice. These results suggest a potential genetic cause for some forms of congenital heart disease in humans.

This summary was initially drafted using artificial intelligence, then revised and fact-checked by the author.

Introduction

Congenital heart disease (CHD) is the most common congenital anomaly in newborns, affecting 8-12/1000 live births worldwide and accounting for approximately 40% of prenatal deaths^1^. CHD arises from abnormal heart development during the embryonic stage and has a strong heritable component. Cardiogenesis is a precise and tightly controlled process involving outflow tract (OFT) formation, the development of four cardiac chambers, and the specialization of cardiac muscle cells^2^. Many signaling pathways and tissue-specific transcriptional regulators contribute to the regulation of heart development. Dysregulation of the dynamic control of these specific biological processes may lead to CHD. For instance, the Notch pathway plays a crucial role in early OFT formation by arresting proliferation and promoting the differentiation of second heart field progenitors^3^, and defects in this pathway lead to various CHD phenotypes^4^.

Super-enhancers (SEs) are a class of regulatory regions comprising clusters of enhancers densely occupied by master regulators and mediators^5^. SEs exert powerful transcriptional activation effects on genes that are crucial for cell identity^6^. Previous evidence suggested that SEs play a significant role in the genetic control network of the heart^7–9^. For example, TGF-β-mediated binding of the chromatin reader protein BRD4 to SEs can promote the expression of genes involved in cardiac fibroblast activation^8^. A conserved SE controls the expression of *Nppa* and *Nppb* in heart development and homeostasis. Deletion of this SE results in manifestations similar to those in *Nppa*-*Nppb* knockout mice, indicating the physiological importance of this SE in cardiac disease^9^. Although a study analyzing a large database generated a comprehensive catalog of SEs in 86 human cells and tissue types, including the left ventricle (LV)^10^, the identification and in-depth analysis of heart-specific SEs remain necessary.

Many SEs are distally related to the genes they control and often regulate their target genes through chromatin loops, which are mediated by cohesin^11^. Cohesin, a ring-shaped protein complex that acts as a chromatin glue, is loaded onto chromatin by NIPBL to form a chromatin loop through DNA extrusion^12^, uniting distant SEs with promoters to coordinate gene expression^13^. Cornelia de Lange syndrome (CdLS, OMIM122470) is a rare genetic disorder resulting from mutations within genes encoding components of cohesin, including NIPBL (65%), HDAC8 (8%), SMC1A (5%) and SMC3 (1–2%)^14–16^. Notably, a considerable proportion of individuals with CdLS also exhibit CHD, which contributes significantly to morbidity and mortality rates^17^. An analysis of cardiac phenotypes in model animals revealed that NIPBL deficiency contributes to heart defects associated with cohesinopathy. *Nipbl*-heterozygous mouse models constructed from Cre expressed in diverse cardiac cell lineages of mouse embryos show atrial septal defects (ASDs) at a high frequency due to subtle global transcriptional dysregulation^18^. *Nipbl* morphant zebrafish exhibit heart jogging/looping defects in the context of abnormal endoderm development^19^. However, the detailed mechanism by which cohesin defects lead to CHD remains unclear, particularly in cases involving mutations in other cohesin components.

In this study, we elucidated the fundamental significance of SMC3 in heart development, as it effectively controls the expression of master genes crucial for cardiogenesis by regulating specific spatial chromatin structures containing SEs. Heart-specific *Smc3*-knockout (SMC3-cKO) mice exhibited varying degrees of OFT malformations, including malformations of the nonseparated aorta and pulmonary artery, transposition of the great arteries (TGA), and membranous ventricular septal defects (VSDs). We also observed 16 rare variants of *SMC3* with potential pathogenicity in patients with isolated CHD, with phenotypes similar to those observed in SMC3-cKO mice. These findings shed light on the essential function of SMC3 in heart development and establish an association between SMC3 and isolated CHD.

## Materials and methods

### RNA scope

An RNA scope assay (Biotend Biotechnology, Shanghai, China) was used to analyze *Smc3* and *Ets2* expression in mouse hearts. Samples were analyzed using the RNA scope 2.5 HD Reagent-Red kit (Advanced Cell Diagnostics, CA, United States) and the RNA scope H2O_2_ & Protease Plus Reagents kit (Advanced Cell Diagnostics, CA, United States). The general processes are described below. Sections were deparaffinized using xylene and 100% ethanol and incubated with a hydrogen peroxide solution for 10 min at room temperature. Afterward, sections were incubated in the target retrieval reagent solution for 15 min at 99 °C, and protease was added for digestion. Sections were hybridized with targeted probes against *Smc3* (Probe-Mm-Smc3-C1) and *Ets2* (Probe-Mm-Ets2-C2). Internal positive controls (against *Polr2a*, *Ppib*, and *Ubc*) and a negative control (against the bacillus gene *DapB*) were used in addition to staining the same tissue to ensure the specificity of the targeted probes. The sections were subjected to signal amplification using AMP1 for 30 min, AMP2 for 30 min, and AMP3 for 15 min and then incubated with the appropriate fluorescent dye. Finally, HRP blockers were added dropwise, and the sections were counterstained with DAPI for 1 min. The prepared sections were sealed with blocking reagents to avoid fluorescence quenching.

### RNA extraction, reverse transcription, and quantitative real-time PCR

Total RNA was extracted from mouse heart tissues and cells using TRIzol reagent (Invitrogen, CA, USA) according to the manufacturer’s instructions. After the evaluation of the quality and concentration, RNA was reverse transcribed to cDNA using a Prime Script RT Reagent Kit (Takara, Shiga, Japan), and quantitative real-time PCR (qPCR) was conducted using SYBR Premix Ex Taq™ (Takara, Shiga, Japan) on a StepOnePlus^TM^ Real-Time PCR System (Thermo Fisher Scientific, MA, USA). The relative expression level of mRNA was normalized to that of GAPDH and calculated using the relative quantification method (2^−ΔΔCt^). The primers used are shown in Supplementary Table 1.

### Construction of heart-specific *Smc3*-knockout mice

*Smc3*^fl/+^ mice (C57BL6/J background) were generated using CRISPR-Cas9 technology at Biomodel Organism Science & Technology Development Co., Ltd., Shanghai, China. The loxp sites flanking exon four were inserted via homologous recombination. *Nkx2.5-Cre* transgenic mice (C57BL6/J background), which express Cre recombinase in their heart-forming fields in the splanchnic mesoderm at embryonic day (E)7.5, were obtained from GemPharmatech, Nanjing, China. The *Smc3*^fl/+^ mice were bred with *Nkx2.5-Cre* mice to generate mice with a deletion of *Smc3* in cardiac progenitors. The genotyping primers used are listed in Supplementary Table 1. The efficiency of *Smc3* knockout in the embryonic heart was tested via qPCR. The embryos were dated, and the morning of the vaginal plug was recorded as E0.5. All transgenic mice were backcrossed to wild-type mice (C57BL6/J background) once after each two to three generations of mating. All mouse procedures were approved and monitored by the Research Ethics Committee of the School of Basic Medical Sciences, Fudan University, China. The approval number is 20160520-3.

### Observation of cardiac phenotypes in mice

Pregnant mice and neonates were anesthetized by isoflurane inhalation and euthanized by rapid cervical dislocation. Embryos and neonates were collected at specific stages and photographed in phosphate-buffered saline (PBS) using a Leica M205C microscope (Leica, Heilbronn, Germany). The ventricular area and compact layer of ventricular myocardium thickness of the mouse hearts were measured using ImageJ (http://rsb.info.nih.gov/ij). Hematoxylin–eosin (H&E) staining was used to observe the detailed anatomical structure of the mouse hearts and was performed by Biossci Biotechnology Co., Ltd., Shanghai, China.

### Clinical subjects and ethics statement

All patients with isolated CHD were recruited from the Children’s Hospital of Fudan University and were diagnosed via echocardiography using published diagnostic criteria. The patients had no other physical abnormalities. A total of 104 healthy Han Chinese individuals recruited from the physical examination department were used as controls. The studies involving human subjects were approved by the Medical Ethics Committee of the Children Hospital of Fudan University, and the approval number is 2016-121.

### Sanger sequencing

Whole blood was collected from 56 patients with isolated CHD, including 34 TOF patients and 22 VSD patients, and 104 Han Chinese controls for Sanger sequencing to detect *SMC3* variants. Sanger sequencing was subsequently performed at the Medical Laboratory of Nantong ZhongKe, Nantong, China. PCR products were sequenced using an ABI 3730 Genetic Analyzer with the respective forward or reverse primers. Chromatograms were analyzed using Mutation Surveyor DNA Variant Analysis Software (https://softgenetics.com/).

### Multiplex PCR-targeting sequencing

The cohort of 1083 patients with isolated CHD^20^ included 234 tetralogy of Fallot (TOF) patients, 718 VSD patients, 55 pulmonary atresia+VSD patients, 19 double outlet right ventricle + pulmonary stenosis patients and 57 right ventricular hypoplasia patients. Multiplex PCR-targeting sequencing was applied to sequence all exons, splice sites (±20 bp of adjacent intronic sequences) and regulatory regions (5000 bp upstream of the transcription start site, 300 bp downstream of the transcriptional termination site) of *SMC3*. The primer design covered a theoretical 99.9% of the complete target region. The prepared samples were sequenced on the HiSeq 2500 (Illumina, California, USA) platform. Burrows‒Wheeler Aligner software (version 0.7.17), SAMtools (https://samtools.sourceforge.net/), and Genome Analysis Toolkit (https://gatk.broadinstitute.org/hc/en-us) were used to map sequence reads to hg19 to calculate the read quality and call single nucleotide variants.

### Identification of *SMC3* variants

Sequence variants were functionally annotated using the ANNOVAR program with annotation databases, including the Reference Sequence (RefSeq, hg19) collection, the pathogenicity prediction databases (the Combined Annotation Dependent Depletion (CADD) (version 1.3), Sorting Intolerant From Tolerant (SIFT) (http://sift-dna.org/sift4g), the polymorphism phenotyping v2 (Polyphen2) (http://genetics.bwh.harvard.edu/pph2/), and reference databases including normal sample sequencing data (the Genome Aggregation Database (GnomAD) (http://gnomad.broadinstitute.org/) and the Exome Aggregation Consortium (ExAC) (http://exac.broadinstitute.org)).

The effect of each screened variant in the promoter and 5’ UTR regions on regulatory DNA elements (DNase hypersensitivity regions, binding sites of transcription factors, and promoter regions) was evaluated via RegulomeDB (https://regulomedb.org/regulome-search/).

For each screened variant at the 3’ UTR, the effect on mRNA–microRNA interactions was predicted using TargetScanHuman 7.0 (http://www.targetscan.org).

Genomnis HSF Pro (https://hsf.genomnis.com/mutation/analysis) was used to evaluate changes in splicing signals and to analyze the effect of variants in introns on consensus splice sites.

The protein sequences of SMC3 from multiple species were obtained from the NCBI database and compared using ClustalW in MEGA11 software (v11.0.13). The evolutionary tree was constructed and tested using the neighbor-joining algorithm and the bootstrap test, respectively. The number of iterations was 1000.

### Dual-luciferase reporter assay

The recombinant luciferase plasmids (pGL3-basic-SMC3-wild-type, pGL3-basic-SMC3-498insTGGGG, and pGL3-basic-778_776delATG) were constructed with the corresponding sequences amplified from human genomic DNA. The plasmids were sequenced and shown to be consistent with the sequences in the National Center for Biotechnology Information database. Cells were seeded in 96-well plates (1 × 10^4^ cells per well) and incubated at 37 °C overnight. The pGL3-basic-SMC3-wild-type, pGL3-basic-SMC3-498insTGGGG, and pGL3-basic-778_776delATG plasmids were transfected into cells using Lipofectamine 3000 (Invitrogen, CA, USA). The cells were collected 48 h after transfection. Both firefly and Renilla luciferase activities were measured using a dual-luciferase reporter assay (Promega, Wisconsin, USA). The firefly luciferase activities were normalized to the Renilla luciferase activities.

### Single-nucleus RNA sequencing

E12.5 mouse embryos were microdissected in PBS solution using a Leica M205C microscope (Leica, Heilbronn, Germany), and the heart tissues were extracted. Whole heart samples from different embryos, including six *Smc3*^fl/fl^ embryos, seven *Smc3*^fl/+^; *Nkx2.5-*Cre embryos, and seven *Smc3*^fl/fl^; *Nkx2.5-*Cre embryos, were subjected to mechanical trituration to prepare cell suspensions. Cell counts and viability were determined for the resulting suspension, and the cell concentration was adjusted to the ideal of 300–600 cells/μL. The prepared single-cell suspension was combined with a mixture of gel beads containing barcode information and enzymes and then encapsulated by oil droplets to form a gel bead-in-emulsion (GEM). After the reverse transcription reaction, the GEMs were fragmented to complete the amplification of nuclear cDNA, and the quality-checked amplification products were constructed as sequencing libraries. Briefly, the cDNA was broken into fragments of 200–300 bp, end-repaired, ligated to the P7 adapter, and finally amplified by PCR for introduction into the indices of the samples. Sequencing libraries were created by integrating the screened cDNA fragments, and single-nucleus RNA sequencing (snRNA-seq) was performed on the NovaSeq 6000 (Illumina, California, USA) platform.

### Single-nucleus RNA sequencing data analysis

Bioinformatics analysis was performed by CapitalBio Technology (Beijing, China). For each sample, the cleaned data were generated using Cell Ranger (v3.0.2) (https://github.com/10XGenomics/cellranger) and filtered for low-quality reads. The data were aligned to the mouse mm10 reference genome. The 10× Genomics‐derived data (*Smc3*^fl/fl^, *Smc3*^fl/+^; *Nkx2.5-*Cre, and *Smc3*^fl/fl^; *Nkx2.5-*Cre) were collected. Downstream analysis using the Seurat (v4.0.5) (https://satijalab.org/seurat) program revealed cells with gene numbers greater than 200, mitochondrial gene ratios less than 25%, and 2000 genes with highly variable expression in 3 or more cells. Principal component analysis was then performed using significantly differentially expressed genes, and uniform manifold approximation and projection (UMAP) was performed to visualize the data. Cells were represented in a two-dimensional UMAP plane, and clusters were identified and annotated according to previously published cardiac cell markers. The significance of differential expression was calculated using the bimod test. The functional enrichment of the differentially expressed genes was then determined by performing Gene Ontology (GO) (http://www.geneontology.org/) analysis. The pseudotime trajectory was determined using the Monocle2 package with the default settings. Specifically, the DDRTree method was used for dimension reduction with max_components set at 2, and the cells were ordered using the orderCells function.

### Construction of *SMC3*-knockdown and *SMC3*-overexpressing myocardial cell lines

The mouse cardiomyocyte cell line HL-1, human cardiomyocyte cell line AC16 and HEK293T cells were cultured in Dulbecco’s modified Eagle’s medium supplemented with 10% fetal bovine serum (FBS) (Gibco, Australia) and 1% Pen-Strep antibiotics (Yeasen, Shanghai, China) at 37 °C in 5% CO_2_.

*SMC3* was knocked down using CRISPR-Cas9. The *SMC3* CRISPR guide RNA (gRNA) was designed using a web tool developed by the Feng Zhang group at MIT and subcloned between the two BsmBI sites of the lentiCRISPRv2 vector from Feng Zhang’s laboratory. The *SMC3* and *Smc3* gRNA target sequences were 5’-CGGCCCTTACCGGCCCATGA-3’ and 5’-CATGCTGTTGAAGAAGAAGG-3’, respectively.

*SMC3* was tagged at the N- and C-termini with an HA tag and then cloned and inserted into the mammalian expression vector pCDH-CMV-IRSE-Blast (pCDH-SMC3-WT). Two variant plasmids (pCDH-SMC3-T857S and pCDH-SMC3-Y434C) were generated using the KOD-Plus Mutagenesis Kit (Toyobo, Tokyo, Japan). The pCDH-ETS2 plasmid (pCDH-CMV-IRSE-Blast) was obtained from GeneRay (Shanghai, China). All expression plasmids were fully sequenced.

For the construction of stable cell lines, ViaFect™ Transfection Reagent (Promega, Wisconsin, USA) was used to package pseudoviral particles of gRNA in HEK293T cells. The virus-containing supernatant was collected, filtered, and used to infect cells in complete media supplemented with 5 µg/mL polybrene (Sigma‒Aldrich, Massachusetts, USA). Puromycin dihydrochloride and blasticidin (Yeasen, Shanghai, China) were used to screen stably transfected *Smc3*-knockdown (KD) and *Ets2*-overexpressing cells, respectively, at 48 hours postinfection.

For the construction of transiently infected cell lines, three plasmids (pCDH-SMC3-WT, pCDH-SMC3-T857S, and pCDH-SMC3-Y434C) diluted to the same concentration (300 ng/μl) were transfected into *SMC3*-KD AC16 cells using the ViaFect™ Transfection Reagent (Promega, Wisconsin, USA). The transfected cells were harvested and used for subsequent experiments 48 hours later.

### Western blotting and antibodies

Cells were harvested and lysed in RIPA buffer (Yeasen, Shanghai, China) containing a protease inhibitor cocktail (Sigma‒Aldrich, Massachusetts, USA). Proteins were quantified using a Bradford Protein Assay Kit (Abcam, MA, USA), and the same amounts of proteins were separated on 10% sodium dodecyl sulfate (SDS)-PAGE gels and transferred to nitrocellulose membranes, which were blocked with 8% milk dissolved in Tris-buffered saline and Tween for 1 h at room temperature. The membranes were then incubated with specific primary antibodies at 4 °C overnight, followed by an incubation with secondary antibodies at room temperature for 1 h. The antibodies used are listed in Supplementary Table 1.

### Bulk RNA-seq and analysis

Bulk RNA-seq of mouse embryo hearts and HL-1 cells was performed by LC-Bio Technology (Hangzhou, China). Total RNA was isolated and purified using TRIzol reagent (Invitrogen, CA, USA). The RNA samples that passed quality control were fragmented into small pieces at 94 °C for 5–7 min and reverse-transcribed to generate cDNA. The average insert size for the final cDNA library was 300 ± 50 bp. The FASTQ sequencing data were trimmed via paired-end sequencing. Consecutively, the trimmed reads were mapped to the reference mouse genome (mm10) with HISAT2 software (https://ccb.jhu.edu/software/hisat2). The mRNA expression abundance was analyzed using StringTie software and represented as FPKM. The differentially expressed mRNAs were selected with FC > 1.5 and a parametric test comparing nested linear models (*p* value < 0.05) using the R package edgeR (https://bioconductor.org/packages/release/bioc/html/edgeR.html). GSEA was performed on all expressed genes that were ranked by log_2_FC value. The functional enrichment of differentially expressed genes was then assessed by performing GO (http://www.geneontology.org/) analysis.

### Chromatin immunoprecipitation assay

HL-1 cells (5–10.0 × 10^6^) were crosslinked with 1% formaldehyde and quenched in a glycine solution (at a final concentration of 0.125 M). Chromatin was prepared as described above and digested with micrococcal nuclease to mainly generate mononucleosomes with a minor fraction of dinucleosomes. The reaction was stopped by the addition of 8 μL of solution containing 0.2 M EDTA and 0.2 M EGTA. The chromatin was incubated overnight at 4 °C with 2 μL of rabbit anti-SMC3 antibody (Abcam, MA, USA), 1 μL of rabbit anti-H3K4me3 antibody (Millipore, Darmstadt, Germany), 1 μL of rabbit anti-H3K27ac antibody (Millipore, Darmstadt, Germany), or 2 μL of control rabbit IgG (CST, MA, USA). Protein A/G magnetic beads (Thermo Fisher Scientific, MA, USA) were added and incubated for an additional 4 h, after which the immunoprecipitates were washed vigorously, and the DNA was purified according to the manufacturer’s instructions for the SimpleChIP® Plus Enzymatic Chromatin IP Kit (CST, MA, USA).

For chromatin immunoprecipitation–sequencing (ChIP-seq), ChIP products were end repaired, dA-tailed and linkers were ligated, and barcoding and Illumina adapter addition were performed by PCR amplification. Libraries were purified using QiaQuick PCR purification reagents (Qiagen, Hilden, Germany), and 0.7× and 0.2× Ampure XP beads (Beckman, California, USA) were used for size selection.

For ChIP‒qPCR, ChIP-enriched DNA samples were quantified by qPCR to determine the binding of SMC3 to the *Ets2* promoter. The values are shown as relative enrichment normalized to that of IgG. The primers used for ChIP‒qPCR are shown in Supplementary Table 1.

### Identification of SEs

SEs were defined by H3K27ac peaks in the mouse cardiomyocyte cell line HL-1 from ChIP-seq data using the rank ordering of SEs (ROSE) algorithm (https://github.com/stjude/ROSE). The details of this procedure were similar to those in previous research^6^. SE-associated genes were screened through a conjoint analysis of transcriptomic data from HL-1 cells. The specific steps were as follows: 1. A total of 1544 genes were filtered because they were most closely related to SEs. 2. Using FPKM values, 308 genes in the top 20% of the 1544 genes were identified as SE-associated genes.

### 3C-high-throughput genome-wide translocation sequencing

A total of 1 × 10^7^ HL-1 cells were subjected to cross-linking by incubating them for 10 min on ice in 10 mL of RPMI-1640 containing 10% FBS and 2% formaldehyde. The reaction was stopped by adding glycine to a concentration of 0.125 M and incubating the mixture for 5 min at room temperature. Pelleted nuclei were incubated for 1 h at 37 °C in 0.5 mL of 1× CutSmart digestion buffer containing 0.3% SDS. The chromatin was then digested by the addition of 400 U of MboI (NEB, MA, USA) overnight at 37 °C, and the reaction was terminated by the addition of 80 μL of 10% SDS. Decrosslinking was performed by adding 30 μL of 10 mg/mL proteinase K and 3 μL of 100 mg/mL RNaseA and incubating the sample for 4 h at 65 °C. A total of 10 μg of 3 C sample was sonicated for 3 min using a Model 550 Sonic Dismembrator (Thermo Fisher Scientific, MA, USA) for 15 s ON and 25 s OFF. As determined by the agarose gel analysis, the chromosomal DNA was reduced to an average size of 300–500 bp. Sonicated DNA was linearly amplified using a biotinylated primer (Supplementary Table 1) that anneals to the selected MboI (NEB, MA, USA) fragment of the *Ets2* promoter. The biotin-labeled single-stranded DNA products were enriched with streptavidin C1 beads (Thermo Fisher Scientific, MA, USA) and ligated with HTGTS adaptors containing a 6-nucleotide overhang. The adaptor-ligated products were amplified using a nested primer that anneals upstream of the biotinylated primer and an HTGTS-adaptor-complementary primer (Supplementary Table 1). The products were prepared for sequencing on the Illumina MiSeq platform (Illumina, California, USA) after amplification using the P5-I5 and P7-I7 primers. After quality control, filtered reads were extracted from the sequence file through Cutadapt and Pear (v0.9.6). Paired-end reads containing NestPrimer or AdapterPrimer were obtained, and then the reads were filtered by searching for restriction site sequences. The first MboI enzyme fragment was extracted and mapped to the mouse genome mm10 using Bowtie2 (version 2.3.4.3). The read numbers were counted and normalized to the total mapped reads per sample after self-ligation, relegation, and dumping. For visualization, we converted the final bam file to a bedGraph file. The signal peak was obtained by postcomparison filtering, signal statistics and normalization.

### Statistical analysis

All experiments were repeated more than twice, and all data are presented as the means±standard deviations. Statistical tests were performed using GraphPad Prism Software (v7). Two-tailed unpaired Student’s *t* tests with or without Welch’s correction or Mann‒Whitney *U* tests were used to analyze the differences between two groups. A *p* value < 0.05 was regarded as statistically significant.

## Results

### Cornelia de Lange syndrome patients with *SMC3* mutations have high rates of congenital heart disease

Mutations in genes encoding components of the cohesin complex are associated with a wide range of effects on heart development in individuals with CdLS^14,21,22^. We conducted an extensive literature search and extracted detailed clinical information for 410 CdLS patients documented in 20 papers to gain deeper insights into the origin of cohesinopathy-related heart defects (Supplementary Table 2). None of these patients were from consanguineous families. An analysis of the data revealed that approximately one-quarter of the CdLS patients exhibited congenital heart defects. The occurrence of CHD was as follows: *NIPBL* mutation carriers (26.73% or 58/217), *RAD21* mutation carriers (25.58% or 11/43), *SMC1A* mutation carriers (24.14% or 14/58), and *HDAC8* mutation carriers (16.33% or 8/49). Notably, patients with *SMC3* mutations had a significantly greater frequency of CHD (52.4% or 22/42) (Supplementary Table 2). These findings suggest that mutations in *SMC3* may have a more pronounced impact on heart development than mutations in other genes encoding cohesin components.

### Heart-specific *Smc3*-knockout mice exhibit defects in outflow tract development

**Fig. 1:**
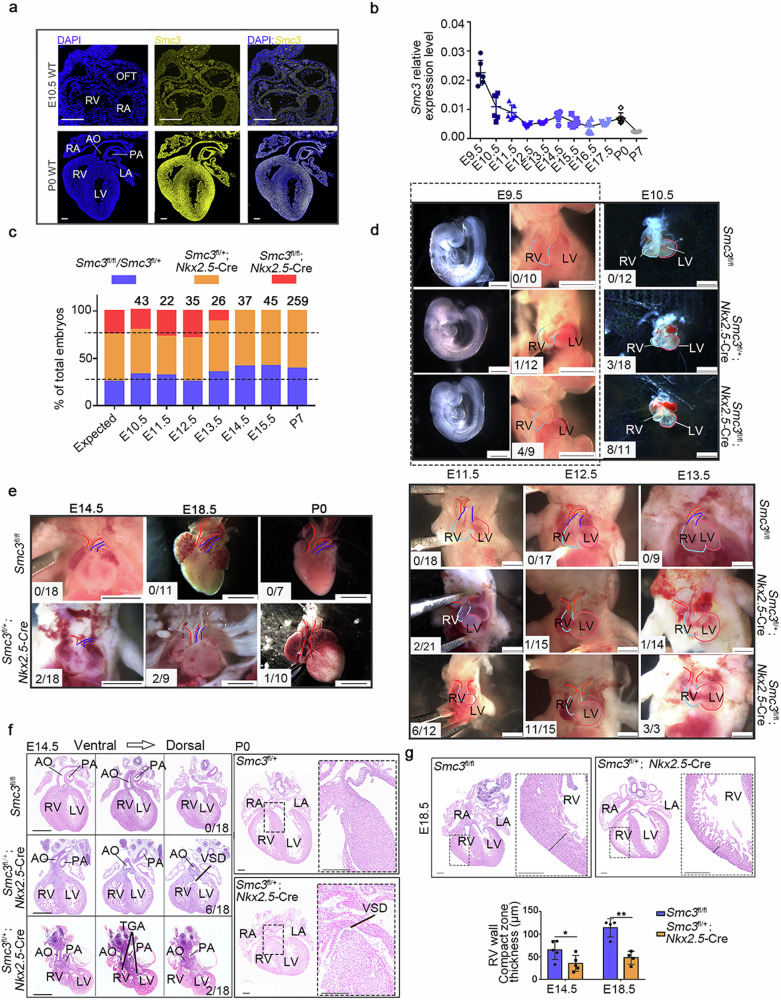
Outflow tract defects in heart-specific *Smc3*-knockout mice. **a** Location of *Smc3* expression in mouse heart at embryonic day (E) 10.5 (top panel) and postnatal day (P) 0 (bottom panel). RNA fluorescence in situ hybridization was performed using the RNA scope platform with probes targeting *Smc3*. Image scale bars, 0.2 mm. **b** Temporal expression pattern of *Smc3* in mouse hearts from E9.5 to P7 determined using quantitative real-time PCR (qPCR). **c** Genotype distribution of embryos and newborn mice resulting from *Smc3*^fl/fl^ and *Smc3*^fl/+^; *Nkx2.5-*Cre crosses. **d** Representative necropsy images of the ventricular size, length of the outflow tract (OFT), and relative location of the aorta (AO) and pulmonary artery (PA) in *Smc3*^fl/fl^*, Smc3*^fl/+^; *Nkx2.5-*Cre, and *Smc3*^fl/fl^; *Nkx2.5-Cre* embryos at E9.5, E10.5, E11.5, E12.5, and E13.5. Image scale bars, 1 mm. **e** Representative images of hearts from fetal and newborn *Smc3*^fl/fl^ and *Smc3*^fl/+^; *Nkx2.5-*Cre mice at E14.5, E18.5, and P0. Compared with *Smc3*^fl/fl^ hearts, *Smc3*^fl/+^; *Nkx2.5-*Cre hearts have different degrees of abnormal arrangement of the large arteries. Image scale bars, 2 mm. **f** H&E staining of heart sections from *Smc3*^fl/fl^ and *Smc3*^fl/+^; *Nkx2.5-*Cre mice at E14.5 and P0. A ventricular septal defect (VSD) was observed in 6/18 *Smc3*^fl/+^; *Nkx2.5-*Cre mice at E14.5. In 2/18 *Smc3*^fl/+^; *Nkx2.5-*Cre mice at E14.5, the PA connects to the left ventricle (LV), and the AO connects to the right ventricle (RV). Scale bars for images of E14.5 embryos, 0.5 mm; scale bars for images of P0 mice, 0.25 mm. **g** H&E staining of heart sections from *Smc3*^fl/fl^ and *Smc3*^fl/+^; *Nkx2.5-*Cre mice at E18.5 (left panel) and quantification of the RV wall compact zone thickness at E14.5 and E18.5 (right panel). The compact layer of the ventricular myocardium is indicated by the black line. Image scale bars, 0.25 mm. The error bars indicate the means ± standard deviations. *P < 0.05 and **P < 0.01. TGA transposition of the great arteries, RA right atrium, LA left atrium.

We detected *Smc3* expression in mouse heart tissues via the RNA scope assay, a recently developed in situ hybridization technique that allows sensitive and specific localization of gene expression in cells and tissues, to clarify the role of SMC3 in cardiac development. *Smc3* was found to be widely expressed across the entire heart at E10.5 and postnatal day (P) 0 (Fig. 1a). *Smc3* was expressed at a relatively high level at E9.5 and decreased at E10.5. Afterward, it maintained a relatively low expression level (Fig. 1b). These results indicate that *Smc3* expression in the heart is widespread and dynamic in early embryonic stages.

Then, we generated SMC3-cKO mice by crossing *Smc3*-floxed (*Smc3*^fl/+^) mice with *Nkx2.5*-Cre mice (Supplementary Fig. 1a, b) and confirmed efficient knockout at E10.5 using qPCR (Supplementary Fig. 1c). The birth rate of the SMC3-cKO mice did not conform to the Mendelian ratio (Fig. 1c, Supplementary Fig. 1d), indicating embryonic lethality. Moreover, *Smc3*^fl/fl^; *Nkx2.5-*Cre embryos were absent at E14.5 (Fig. 1c, Supplementary Fig. 1d). During heart looping (E9.5 to E10.5), a significant proportion (12/20) of *Smc3*^fl/fl^; *Nkx2.5-*Cre embryos exhibited a short OFT and small right ventricle (RV) compared to *Smc3*^fl/fl^ embryos (Fig. 1d). During normal heart development, the OFT is separated into the aorta and pulmonary artery from E11.5 to E13.5. However, we did not observe a tendency toward OFT separation in 6 of the 12 *Smc3*^fl/fl^; *Nkx2.5-*Cre embryos at E11.5, and the malformation rate increased to 73.3% (11/15) at E12.5. All surviving *Smc3*^fl/fl^; *Nkx2.5-*Cre embryos presented this type of abnormality, especially at E13.5 (Fig. 1d), which may have led to their death after this time point. Considering the dual roles of cohesin in embryonic development^23,24^, we propose that the embryonic lethality in *Smc3*^fl/fl^; *Nkx2.5-*Cre embryos may be attributed to defective cell proliferation.

On the other hand, *Smc3* heterozygous mutations have been associated with abnormal cardiac development in CdLS patients, possibly resulting from the dysregulated expression of genes involved in heart development^24^. We analyzed the phenotype of *Smc3*^fl/+^; *Nkx2.5-*Cre embryos to investigate this outcome further. Among the total number of embryos examined (80), only a small proportion (8/80) exhibited OFT developmental defects (Fig. 1d). Surviving *Smc3*^fl/+^; *Nkx2.5-*Cre embryos at E14.5, E18.5, and P0 showed malalignment of the aorta and pulmonary artery, along with small RVs (Fig. 1e). At E14.5, 11% (2/18) of *Smc3*^fl/+^; *Nkx2.5-*Cre embryos exhibited TGA, while 33% (6/18) displayed a membranous VSD (Fig. 1f). Furthermore, at P0, one of ten *Smc3*^fl/+^; *Nkx2.5-*Cre mice had a VSD (Fig. 1f). In addition to structural abnormalities, we noticed a thinner compact layer of the ventricular myocardium in *Smc3*^fl/+^; *Nkx2.5-*Cre embryos at E14.5 and E18.5 (Fig. 1g). Notably, cohesin haploinsufficiency has a mild impact on cell proliferation^25^. Therefore, the observed malformations in *Smc3*^fl/+^; *Nkx2.5-*Cre mice likely resulted from the dysregulation of genes related to heart development. Notably, due to the early embryonic lethality of SMC3-cKO embryos, we were unable to ascertain the extent of SMC3-dependent developmental progress. Consequently, we cannot rule out structural malformations of the LV as a potential cause for incomplete embryos.

### *SMC3* variants are identified in patients with isolated congenital heart disease

**Fig. 2:**
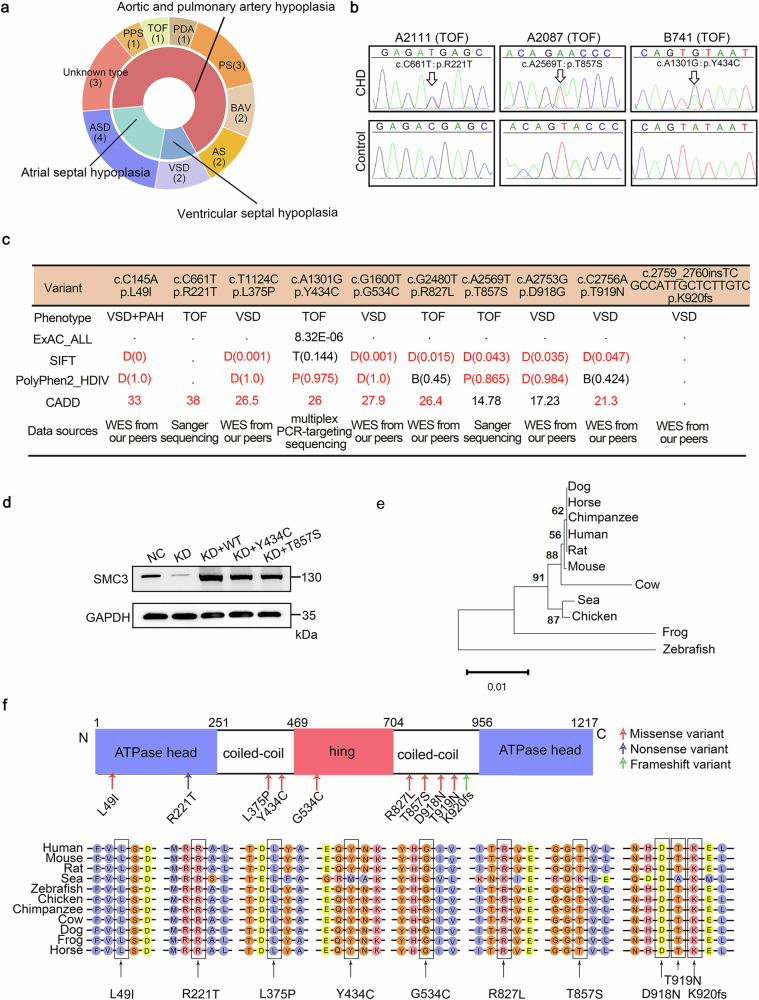
Analysis of *SMC3* variants in patients with isolated congenital heart disease. **a** Statistical overview of the types of congenital heart disease (CHD) in Cornelia de Lange syndrome (CdLS) patients with *SMC3* mutations. The inner layer represents the pathological categorization of CHD types. The outer layer shows CHD types. The digit in brackets indicates the number of CdLS patients. **b** Sanger sequencing chromatograms of three *SMC3* coding variants (R221T, T857S, and Y434C). The *SMC3* variant (Y434C) was screened by multiplex PCR-targeting sequencing and verified by Sanger sequencing. The black arrows in the upper panels indicate variant sites. The lower panels show wild-type (WT) sequences. **c** General information on the ten coding variants. Red markings represent increased pathogenicity. See Supplementary Table 3 for further details. Categorical predictions from each algorithm. ExAC contains high-quality exome sequencing data for 60,706 individuals from different ancestries. The values represent the frequencies of variant occurrence. SIFT, “T”=tolerated, “D”=damaging. The numbers represent the probabilities of similarity between the variant and the actual variant as calculated using the SIFT algorithm, with values less than 0.05 indicating significant differences. PolyPhen2 HDIV, “B”=benign, “P”=possibly damaging, “D”=likely damaging. Numbers represent the probabilities that the variant changes the protein structure and function as calculated using the HumDiv algorithm. CADD, the numbers represent the score of the deleteriousness of the variant based on several factors, including the polymorphism of the allele and the pathogenicity of the variant, with a higher value representing a greater probability that the variant is “deleterious”. A phred score >20 is generally considered “deleterious”. **d** The effects of variants on SMC3 expression. The level of SMC3 in *SMC3*-knockdown (KD) AC16 cells overexpressing *SMC3*-WT was higher than that in cells overexpressing the variant. **e** Evolutionary tree of SMC3 in eleven species. Each node represents a taxon. The numbers indicate the reliability of the branch, and a larger value indicates greater reliability. The branch lengths represent the genetic variability of the sequence and are calculated based on a distance scale. **f** Protein domain plot of SMC3 and the site (top panel) and conserved region (bottom panel) of ten coding variants. The color of each arrow pointing to the variant represents the variant type. All identified *SMC3* variants are indicated at the corresponding positions. TOF tetralogy of Fallot, PDA patent ductus arteriosus, PS pulmonary stenosis, BAV mitral and aortic valve, AS aortic stenosis, ASD atrial septal defect, PPS peripheral pulmonary stenosis, PAH pulmonary arterial hypertension, NC normal control cells transfected with a blank vector, ExAC exome aggregation consortium, SIFT Sorting Intolerant From Tolerant, Polyphen2 polymorphism phenotyping v2, HDIV HumDiv model, CADD combined annotation-dependent depletion, WES whole-exon sequencing.

De novo mutations in *SMC3* contribute to approximately 1–2% of CdLS-like phenotypes, with a significant proportion displaying noncanonical CdLS phenotypes^14^. Upon examination of the CHD type of CdLS individuals^26–31^ with *SMC3* mutations, we found that a majority (76.2% or 16 out of 21) exhibited aorta and pulmonary artery dysplasia, consistent with the phenotypes of SMC3-cKO mice (Fig. 2a). We extracted DNA samples for Sanger sequencing from 56 individuals with isolated CHD, including those with TOF and VSD, corresponding to the heart defects observed in SMC3-cKO mice, to understand the relevance of *SMC3* variants in CHD patients without a distinct CdLS phenotype. Two candidate pathogenic variants (R221T and T857S) in the coding region were identified in two TOF patients based on the following filtering criteria: 1) excluding synonymous variants; 2) variants with a minor allele frequency (MAF) less than 0.1% according to the public control database; and 3) the variant type was a nonsense, frameshift or missense variant predicted to be deleterious by at least two algorithms (Fig. 2b, c, Supplementary Table 3). Subsequently, through multiplex PCR-targeting sequencing, we searched for *SMC3* variants in a larger cohort of 1083 patients with isolated CHD from our previous study^20^ and found one candidate pathogenic variant (Y434C) in the coding region from a patient with TOF after filtering using the same criteria. We also detected six variants with potential pathogenicity in the noncoding region from patients with VSD or TOF according to a MAF less than 0.1%, and these variants are predicted to affect SMC3 expression (located on regulatory DNA elements, affect the consensus splice site, or influence microRNA binding) (Supplementary Table 3). Furthermore, seven *SMC3* candidate pathogenic variants in the coding region were screened from our peers’ whole exome sequencing data related to TOF or VSD without known pathogenic factors (Fig. 2c, Supplementary Table 3). In summary, a total of 16 *SMC3* candidate pathogenic variants (ten coding and six noncoding) were detected across 14 patients with TOF or VSD. The ten coding variants, including eight missense variants, one nonsense variant, one frameshift variant, and three other variants (R221T, Y434C, and T857S) were verified by Sanger sequencing (Fig. 2b) due to the availability of DNA samples. We also noticed that the missense variants (L49I and R221T) are located in the ATPase head domain, and one (G534C) is in the hinge domain of SMC3 (Fig. 2f). Then, wild-type or mutated *SMC3* was overexpressed in the *SMC3*-KD cardiomyocyte cell line AC16 to explore the effects of these variants on protein expression. We found that two variants (Y434C and T857S) led to a reduction in SMC3 expression at the protein level (Fig. 2d). Furthermore, the evolutionary tree of eleven species indicated that SMC3 is highly conserved among mammalian species (Fig. 2e). The high conservation of these variants across multiple species illustrated their constant and significant role during species evolution (Fig. 2f). We also performed Sanger sequencing on DNA samples from 104 Han Chinese controls to further verify the rarity of these variants and did not find any of these *SMC3* variants through a sequence analysis (Supplementary Fig. 2a).

The cardiomyocyte cell line AC16 was transfected with luciferase reporter plasmids containing the wild-type *SMC3* promoter and two mutant *SMC3* promoters (498insTGGGG and -778_-776delATG) to investigate the impact of noncoding variants on *SMC3* transcription (Supplementary Fig. 2b). Dual-luciferase reporter assays revealed that the two variants reduced promoter activity, suggesting their potential roles in downregulating *SMC3* expression. Additionally, qPCR analysis revealed significantly downregulated *SMC3* expression in injured heart tissues from TOF patients compared to normal heart tissues, the cDNAs of which were obtained from the remaining preserved samples from previous research projects^32^ (Supplementary Fig. 2c). Taken together, these findings suggest an association between *SMC3* variants and isolated CHD.

### The deletion of *SMC3* resulted in altered expression profiles of master genes in cardiac muscle cells

**Fig. 3:**
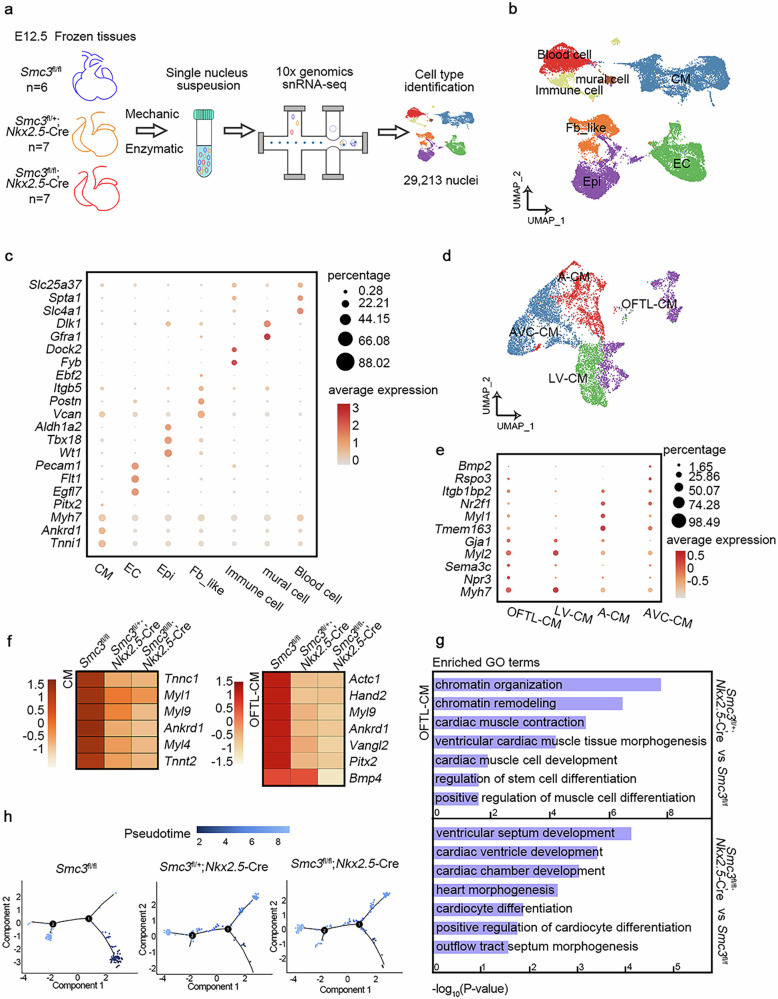
Single-cell profiling of heart-specific *Smc3*-knockout embryonic cardiac tissue at E12.5. **a** Schematic of single-nucleus RNA-seq (snRNA-seq). Uniform manifold approximation and projection (UMAP) representation of single-cell transcriptomes derived from E12.5 *Smc3*^fl/fl^*, Smc3*^fl/+^; *Nkx2.5-*Cre, and *Smc3*^fl/fl^; *Nkx2.5-Cre* embryonic cardiac tissue (**b**) and CMs (**d**). Dot plots showing the expression of individual marker genes across the cardiac tissue (**c**) and CM (**e**) lineages. The color from dark to light represents the average expression from high to low. The size of the dot represents the percentage of cells within that cluster expressing the indicated gene. **f** Heatmap illustrating scaled expression of CM differentiation master genes in CMs and OFT development master genes in outflow tract like (OFTL)-CMs from E12.5 *Smc3*^fl/fl^*, Smc3*^fl/+^; *Nkx2.5-*Cre, and *Smc3*^fl/fl^; *Nkx2.5-Cre* cardiac tissue. The color from dark to light indicates the scaled expression from low to high. **g** Bar plots displaying representative Gene Ontology (GO) terms in which downregulated genes were enriched in OFTL-CMs from E12.5 *Smc3*^fl/+^; *Nkx2.5-*Cre (top panel) and *Smc3*^fl/fl^; *Nkx2.5-Cre* (bottom panel) cardiac tissues. **h** Pseudotemporal ordering trajectory map of OFTL-CMs from *Smc3*^fl/fl^*, Smc3*^fl/+^; *Nkx2.5-*Cre, and *Smc3*^fl/fl^; *Nkx2.5-Cre* embryonic cardiac tissues. Dark to light colors represent the pseudotime order. Fb_like fibroblast-like cell, Epi epicardial cell, EC endothelial cell, A-CM atrium-cardiac muscle cell, LV-CM left ventricle-cardiac muscle cell, AVC-CM atrioventricular canal-cardiac muscle cell.

We focused on E12.5, a stage in which SMC3-cKO mice do not exhibit embryonic lethality and OFT separation begins, to obtain insights into the precise cellular and molecular events driven by SMC3 during heart development. Compared with other organs, the heart mainly contains cardiomyocytes, which are large and single cells cannot be easily captured via microfluidics; therefore, we performed snRNA-seq^33,34^ on cardiac tissues derived from *Smc3*^fl/fl^, *Smc3*^fl/+^; *Nkx2.5-*Cre and *Smc3*^fl/fl^; *Nkx2.5-*Cre mouse embryos at E12.5 using the 10× Genomics technique, generating 8923, 10,057, and 10,233 nuclei, respectively (Fig. 3a). All SMC3-cKO embryonic hearts used for sequencing showed defects in OFT development. Normalized snRNA-seq profiles at the *Smc3* locus in *Smc3*^fl/fl^ and SMC3-cKO embryo cardiac tissue further validated the knockout efficiency (Supplementary Fig. 3a).

The cells were clustered into 27 types based on marker gene expression (Supplementary Fig. 3b), and specific cell types were assigned using the cardiac muscle cell (CM) marker genes *Myh6* and *Ankrd1*, endothelial cell (EC) genes *Pecam1* and *Flt1*, and the epicardial cell (Epi) genes *Wt1* and *Tbx18* (Fig. 3b, c, Supplementary Fig. 3c, d). Additionally, we identified fibroblast-like cells (Fb_like) and mural cells based on the expression of *Postn* and *Dlk1*, respectively (Fig. 3b, c). Immune and blood cells were distinguished by the expression of *Dock2* and *Slc4a1* (Fig. 3b, c). Our particular focus was on CMs, as disruptions in their differentiation or function can cause arrested OFT remodeling and separation, diminished pump function, arrhythmias and ultimately death^35^. Compared with CMs from *Smc3*^fl/fl^ mice, 2129 down-regulated genes were detected in homozygous (*Smc3*^fl/fl^; *Nkx2.5-*Cre) embryonic hearts (Supplementary Table 4) and 2173 downregulated genes were detected in heterozygous (*Smc3*^fl/+^; *Nkx2.5-*Cre) embryonic hearts (Supplementary Table 4). We observed a notable reduction in the expression of critical CM developmental genes, including *Tnnt2* and *Tnnc1*, as well as CM marker genes such as *Ankrd1* and *Myl1/4/9* (Fig. 3f) in SMC3-cKO embryos. GO analysis revealed the enrichment of these downregulated genes in biological processes involved in CM contraction and differentiation (Supplementary Fig. 3e, f). We also identified different CM subtypes based on previously established markers for embryonic CM heterogeneity^36,37^, including OFT-like CMs (OFTL-CMs), atrial CMs (A-CMs), CMs of the left ventricle (LV-CMs), and the atrioventricular canal (AVC-CMs) (Fig. 3d, e, Supplementary Fig. 3g, h).

We performed a comprehensive analysis of the differentially expressed genes in OFTL-CMs from *Smc3*^fl/+^; *Nkx2.5-*Cre or *Smc3*^fl/fl^; *Nkx2.5-*Cre embryos to further explore the underlying cause of abnormal OFT development in SMC3-cKO embryos. Our analysis revealed 4577 downregulated genes in *Smc3*^fl/fl^; *Nkx2.5-*Cre embryonic hearts (Supplementary Table 4) (Fig. 3f), while 4628 genes were downregulated in *Smc3*^fl/+^; *Nkx2.5-*Cre embryonic hearts compared with *Smc3*^fl/fl^ hearts (Supplementary Table 4) (Fig. 3f). GO analysis highlighted enrichment of the downregulated genes in biological processes related to cardiac muscle cell differentiation and heart processes, including OFT morphogenesis (Fig. 3g). Given the similarity in the number and function of differentially expressed genes between *Smc3*^fl/+^; *Nkx2.5-*Cre and *Smc3*^fl/fl^; *Nkx2.5-*Cre embryos at this stage, a plausible hypothesis is that the lethality of homozygous embryos at later stages can be attributed more to the impact of SMC3 on the cell cycle.

As a method to understand whether the underlying differentiation process of OFTL-CMs was affected in each sample, we utilized the R package monocle to construct cell development trajectories. The OFTL-CMs in *Smc3*^fl/fl^ embryonic hearts were predominantly distributed during early and late differentiation, whereas in SMC3-cKO embryonic hearts, more OFTL-CMs were detected in the transition state (Fig. 3h). These results indicate that SMC3 plays a key role in the development and differentiation of CMs, particularly CMs located in the OFT.

Due to the heterogeneity of tissues and the indispensable role of SMC3 in mitosis, we performed bulk RNA-seq to verify the function of SMC3 in cardiac development using the *Smc3* KD mouse cardiac muscle cell line HL-1 (Supplementary Fig. 4a, b). A total of 730 significantly downregulated genes were identified in the KD group compared to the normal control (NC) group (Supplementary Table 5). GO enrichment analysis and gene set enrichment analysis (GSEA) revealed that these genes were enriched in biological processes involved in cardiac morphogenesis and cardiac muscle tissue development (Supplementary Fig. 4c, d). Among the downregulated genes were several established cardiac regulatory genes, including *Tbx1, Notch2*, *Tbx18*, *Ankrd1*, *Agt*, *Mef2a*, and *Ets2* (Supplementary Fig. 4e). These results are consistent with the findings from the snRNA-seq data.

### SMC3 regulates multiple heart development-related genes by controlling the activity of superenhancers

**Fig. 4:**
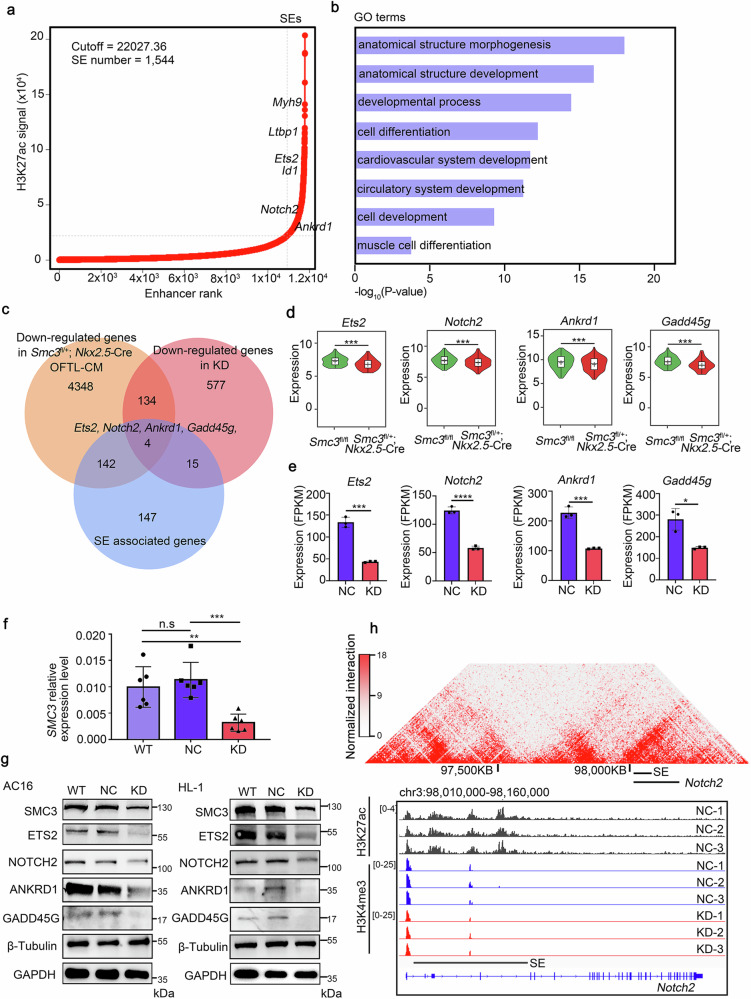
SMC3 controls heart development-related genes through the regulation of super-enhancer functions. **a** Hockey-stick plots illustrating superenhancers (SEs) based on normalized H3K27ac signals and their ranks in the mouse cardiomyocyte cell line HL-1 using the rank ordering of superenhancers (ROSE) algorithm. Selected SE-associated genes are marked. **b** Bar plots displaying representative GO terms enriched in 308 SE-associated genes. **c** Comparison of 4628 downregulated genes in OFTL-CMs from *Smc3*^fl/+^; *Nkx2.5-*Cre embryos, 730 downregulated genes in *Smc3-*KD HL-1 cells, and 308 SE-associated genes. Four overlapping genes, namely, *Ankrd1*, *Gadd45g*, *Ets2*, and *Notch2*, were identified. **d** Violin plots illustrating reduced expression of the four overlapping genes in OFTL-CMs from *Smc3*^fl/+^; *Nkx2.5-*Cre embryos compared to *Smc3*^fl/fl^ embryos. **e** Bar graphs showing the expression of the four overlapping genes in the KD group compared to the NC group. **f** qPCR analysis validated the *SMC3* knockdown efficiency in AC16 cells. **g** Western blot (WB) assays confirmed that SMC3 regulated the protein levels of ETS2, NOTCH2, ANKRD1 and GADD45G in both AC16 (left panel) and HL-1 (right panel) cells. **h** In situ Hi-C chromatin interaction heatmaps of LV cardiac muscle cells obtained from adult WT mice at the *Notch2* locus and SE. H3K4me3 and H3K27ac ChIP-seq tracks of the NC and KD groups at the *Notch2* locus and SE. All error bars are the means ± standard deviations. *P < 0.05; **P < 0.01; ***P < 0.001; and ****P < 0.0001. FPKM, fragments per kilobase of exon model per million mapped fragments.

SEs are known to be highly tissue-specific and are often associated with pivotal regulatory genes involved in cell fate determination, embryonic development, and tumor growth^38–40^. Cohesin, with SMC3 as a core component, regulates gene expression by mediating chromatin organization and enhancer activity^41^. We speculated that SMC3 is involved in heart development by regulating heart-specific SEs. We performed ChIP-seq targeting histone H3 lysine 27 acetylation (H3K27ac) in HL-1 cells to characterize the SE landscape of the heart. Using the ROSE algorithm^5^, we identified 1544 SEs and 308 SE-associated genes (Fig. 4a) (Supplementary Table 6). Some of these SE-associated genes, including *Ets2*, *Myh9*, *Id1*, *Notch2*, and *Ankrd1*, are recognized as master regulatory genes of cardiogenesis in mice (Fig. 4a). GO analysis revealed significant enrichment of these SE-associated genes in biological processes related to anatomical structure development and cell differentiation (Fig. 4b).

Next, we examined the expression of these SE-associated genes in OFTL-CMs from *Smc3*^fl/+^; *Nkx2.5-*Cre embryos, as well as in *Smc3* KD HL-1 cells. Among the 308 SE-associated genes, four genes, *Ankrd1*, *Gadd45g*, *Ets2*, and *Notch2*, were found to be significantly downregulated under both in vivo and in vitro conditions of *Smc3* disruption (Fig. 4c–e). Additionally, we observed that *SMC3* downregulation led to reduced expression of these proteins in *SMC3* KD HL-1 and AC16 cells (Fig. 4f–g). Importantly, these genes are known to play roles in heart development. For instance, Notch2, a receptor involved in canonical Notch signaling, is crucial for proper OFT development^42^. Hi-C data from LV cardiac muscle cells in adult mice (GSE96692) also revealed substantial interactions between *Notch2* and its SE in a topologically associating domain (TAD) according to our reanalysis (Fig. 4h). ETS2 is a transcription factor belonging to the ETS family known for its critical roles in regulating networks that govern the development of the heart, especially the coronary arteries and myocardial tissue^43,44^. However, the mechanisms underlying the regulation of its expression remain elusive. These findings suggest that SMC3 plays a critical role in controlling heart development-related genes by regulating the activity of SEs.

### A super-enhancer regulates *Ets2* expression in the heart

**Fig. 5:**
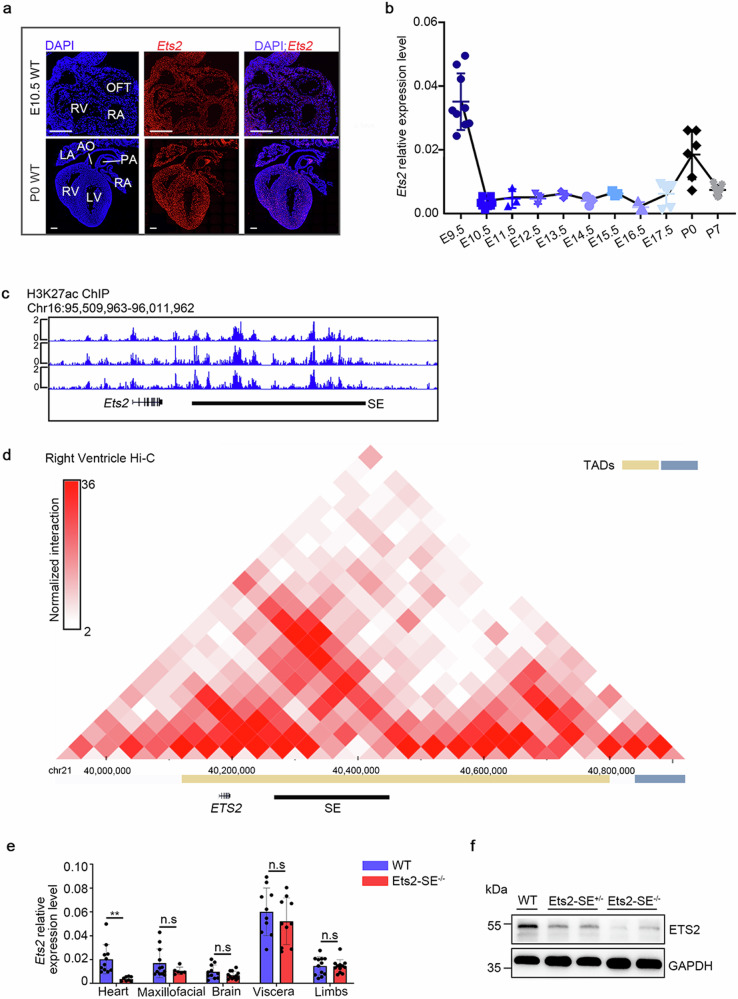
Super-enhancers regulate *Ets2* expression in the heart. **a** Localization of *Ets2* in the hearts of WT mice at E10.5 (top panel) and P0 (bottom panel). RNA fluorescence in situ hybridization images were obtained using the RNA scope platform with probes targeting *Ets2*. Image scale bars, 0.2 mm. **b** Temporal expression pattern of *Ets2* in mouse hearts from E9.5 to P7 analyzed using qPCR. **c** Normalized H3K27ac ChIP-seq profiles at both the *Ets2* locus and SE in HL-1 cells. **d** In situ Hi-C chromatin interaction heatmap of human RV at the *ETS2* locus and SE. **e**, **f** qPCR and WB results illustrating that Ets2-SE deletion specifically reduces *Ets2* expression in the heart at E10.5. All error bars are the means ± standard deviations. **P < 0.01. TAD topologically associating domain.

Demonstrating that the Ets2 super-enhancer (Ets2-SE) indeed promotes *Ets2* expression during cardiac development is crucial to further confirm the role of SMC3 in regulating *Ets2* expression. First, the spatiotemporal expression pattern of *Ets2* in mouse hearts from E9.5 to P7 was determined using an RNA scope assay. We found that *Ets2* was ubiquitously expressed in the heart (Fig. 5a). A temporal analysis of wild-type mice indicated that the *Ets2* mRNA was expressed at its highest level at E9.5 and remained at a relatively low level until a second expression peak at P0 (Fig. 5b), which closely resembles the spatial and temporal specificity of *Smc3* expression in the heart during mouse embryonic development. We also observed a region with strong H3K27ac signals at the Ets2-SE in the ChIP-seq data from HL-1 cells (Fig. 5c). This region largely overlaps with the position of the identified Ets2-SE in thymocytes^6^. Further analysis of ChIP-seq data from human heart tissue (GSM409307, GSM706848, GSM906404, GSM1059445, GSM663427, GSM906396, GSM1013124, GSM432392, GSM772735, and GSM906406) confirmed the presence of an enhancer cluster at the conserved region in the genome (Supplementary Fig. 5a), suggesting that the ETS2-SE is conserved in humans and mice. Furthermore, a robust interaction between this SE and the *ETS2* promoter was observed in RV tissues (GSE87112) (Fig. 5d), indicating the regulatory role of ETS2-SE in regulating *ETS2* expression. Our existing Ets2-SE-deficient mice, described in our previous publication^6^, were analyzed to investigate the impact of Ets2-SE on heart development and *Ets2* expression. In these mice, the deletion of a 166 kb genomic region (chr16:95745432-95912361, mm10) containing Ets2-SE led to a specific approximately 5-fold reduction in ETS2 expression in the heart, as demonstrated by qPCR and WB assays (Fig. 5e, f). Interestingly, despite its proximity to the Ets2-SE and location in the same TAD, the expression of *Psmg1* was not affected by Ets2-SE deletion in the cardiac tissues of mouse embryos at E10.5 (Supplementary Fig. 5b). These results indicate that Ets2-SE specifically regulates the high expression of *Ets2* in the heart.

### SMC3 promotes the transcription of *Ets2* by enhancing promoter–enhancer interactions

**Fig. 6:**
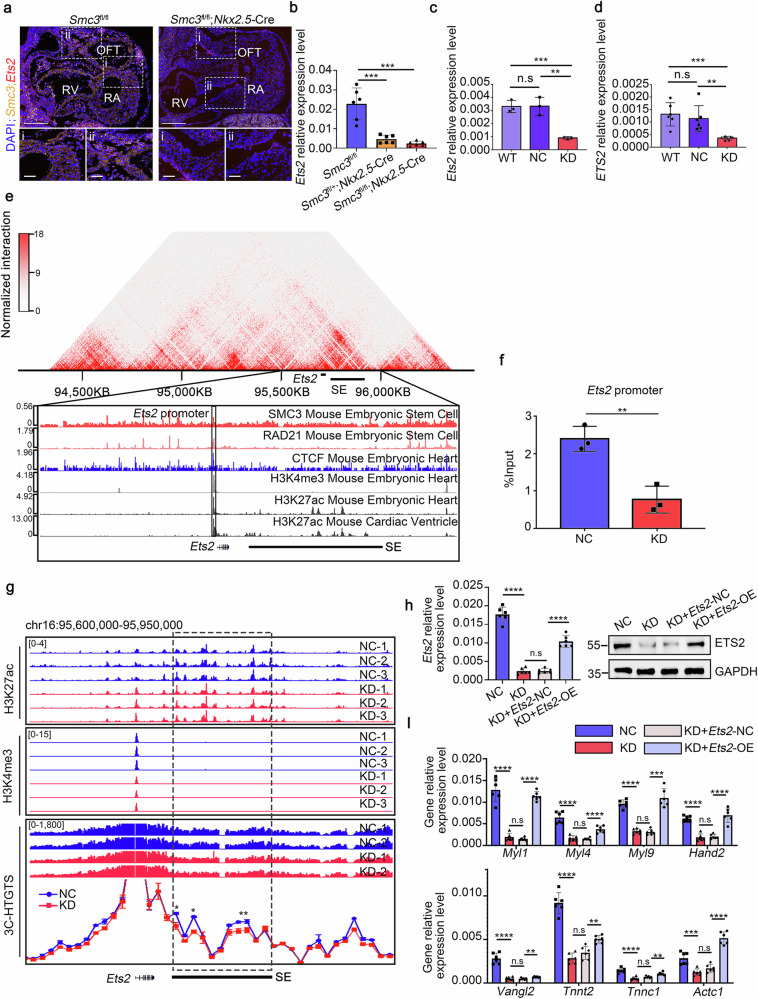
SMC3 regulates *Ets2* expression by mediating promoter–enhancer interactions. **a** RNA fluorescence of mouse embryonic hearts revealed reduced expression of *Ets2* and *Smc3* in *Smc3*^fl/fl^; *Nkx2.5-*Cre mice compared to *Smc3*^fl/fl^ mice. Image scale bars, 0.1 mm. Low-power image scale bars, 0.25 mm; high-magnification image scale bars, 50 μm. **b** qPCR results for the *Ets2* mRNA extracted from the embryonic hearts of *Smc3*^fl/fl^, *Smc3*^fl/+^; *Nkx2.5-*Cre, and *Smc3*^fl/fl^; *Nkx2.5-*Cre mice at E10.5. qPCR analysis of the impact of *SMC3* knockdown on *ETS2* expression in HL-1 (**c**) and AC16 cells (**d**). **e** In situ Hi-C chromatin interaction heatmaps of LV cardiac muscle cells obtained from adult WT mice at the *Ets2* locus and SE. SMC3, RAD21, CTCF, H3K4me3, and H3K27ac ChIP-seq tracks of mouse embryonic stem cells and mouse embryonic hearts for the *Ets2* locus and SE. **f** ChIP‒qPCR revealed reduced occupancy of SMC3 on the *Ets2* promoter in KD HL-1 cells compared to NC HL-1 cells. **g** ChIP-seq and 3C-HTGTS-seq at the *Ets2* locus and SE. The 3C-HTGTS bait is located at the *Ets2* promoter. **h** qPCR and WB analyses validated the efficiency of *Ets2* overexpression (OE) in *Smc3*-KD HL-1 cells. **i** The expression of the genes downregulated in Fig. 3f in four groups of HL-1 cells, namely, NC, KD, overexpression of the blank vector in *Smc3*-KD HL-1 cells (KD + *Ets2*-NC), and overexpression of *Ets2* in *Smc3*-KD HL-1 cells (KD + *Ets2*-OE). The reduced expression of these genes in *Smc3*-KD cells was reversed by *Ets2* overexpression. All error bars are the means ± standard deviations. *p < 0.05, **p < 0.01, ***P < 0.001, and ****P < 0.0001.

We detected *Smc3* and *Ets2* expression in the heart tissues of mouse embryos at E10.5 by performing RNA scope and qPCR assays to explore the regulatory effect of SMC3 on *Ets2* expression during cardiac development (Fig. 6a, b). The results indicated a notable reduction in the fluorescence intensity of the *Ets2* probe across all regions of heart in the *Smc3*^fl/fl^; *Nkx2.5-*Cre embryos (Fig. 6a), which was consistent with the trend detected using qPCR (Fig. 6b). These results indicate that the positive regulatory effect of SMC3 on *Ets2* expression is widespread throughout cardiac tissues.

We used *Smc3* KD HL-1 and AC16 cells to elucidate the mechanism by which SMC3 regulates *Ets2* expression (Supplementary Fig. 4a, b, Fig. 4f, g). The downregulation of *SMC3* led to a reduction in ETS2 expression at both the mRNA and protein levels in mouse and human cell lines (Figs. 4f, g and 6c, d), consistent with observations in *Smc3*^fl/fl^; *Nkx2.5-*Cre mouse heart tissues. Many studies have provided substantial evidence supporting the concept that the loop extrusion model serves as the foundation for cohesin-mediated control of gene expression^45–47^. According to this model, one or a pair of physically tethered cohesin rings slide in opposite directions along chromatin until they encounter hindrance from CTCF proteins bound to inward-oriented motifs. Then, we analyzed the ChIP-seq data for SMC3 (GSM2111724), RAD21 (GSM591469), CTCF (GSM851286), H3K4me3 (GSM1944082), and H3K27ac (GSM2577068 and GSM851290) in mouse embryonic stem cells and heart tissues. High occupancy of SMC3, RAD21, and CTCF was detected on the promoter region of *Ets2* (Fig. 6e), suggesting that cohesin directly regulates *Ets2* expression. ChIP‒qPCR confirmed the binding of SMC3 to the *Ets2* promoter in HL-1 cells, and SMC3 occupancy was reduced in *Smc3*-KD HL-1 cells (Fig. 6f). Additionally, we performed ChIP-seq of H3K27ac and H3K4me3 in HL-1 cells to detect the effect of *Smc3* downregulation on the SE. We observed reduced H3K27 acetylation in the SE region and decreased H3K4 trimethylation at the *Ets2* promoter in *Smc3* KD cells (Fig. 6g).

The role of cohesin in facilitating interactions between promoter regions and distant enhancers, thereby activating gene transcription, is widely recognized^5,41,48^. We conducted an analysis using Hi-C data obtained from LV cardiac muscle cells of adult mice (GSE96692) to explore the involvement of SMC3 in the enhancer–promoter looping of the *Ets2* gene. This analysis revealed strong interactions between the *Ets2* gene and the SE region. We further explored this relationship in *Smc3* KD and NC HL-1 cells utilizing a newly developed technique called 3C-high-throughput genome-wide translocation sequencing (3C-HTGTS-seq) with bait at the *Ets2* promoter. 3C-HTGTS is a technique similar to the circularized chromatin conformation capture (4 C) assay, enabling the sensitive detection of regions interacting with the *Ets2* promoter^6^. We observed that the *Ets2* promoter strongly interacted with the SE region, and these interactions were significantly reduced in *Smc3* KD cells (Fig. 6g). These findings suggest that SMC3 promotes *Ets2* transcription by enhancing SE activity and mediating interactions between the *Ets2* promoter and the SE region.

We overexpressed *Ets2* in *Smc3*-KD HL-1 cells (Fig. 6h) and assessed the expression levels of the downregulated genes (Fig. 3f) in CMs and OFTL-CMs from SMC3-cKO embryos to verify whether SMC3 regulates the expression of cardiac development-related genes through *ETS2*. We found that *Ets2* overexpression significantly reduced the expression of the majority of genes in *Smc3*-KD cells (Fig. 6i, Supplementary Fig. 6). In conclusion, these results indicate that SMC3 regulates *Ets2* expression by establishing chromatin loops between Ets2-SE and its promoter, thereby influencing the regulation of cardiac development-related genes.

### The *Ets2* super-enhancer is involved in the regulation of heart development

**Fig. 7:**
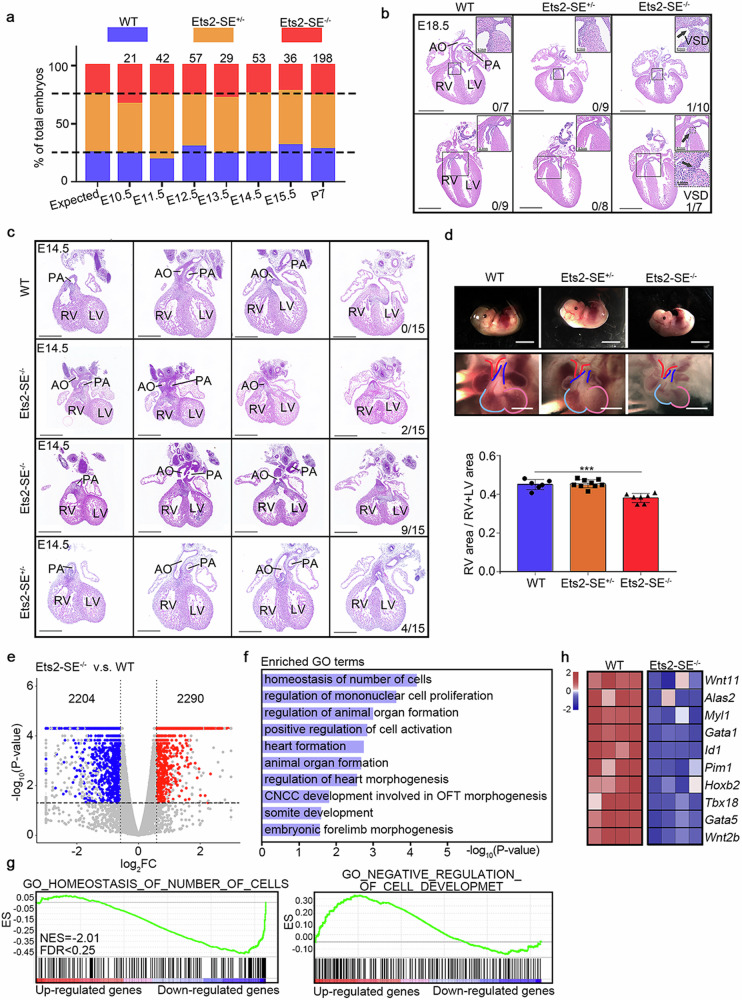
*Ets2* super-enhancer deletion delayed OFT development in mice. **a** Genotype distribution of embryos and newborn mice resulting from Ets2-SE^+/−^ and Ets2-SE^+/−^ crosses. **b** H&E staining of heart sections from WT, Ets2-SE^+/−^, and Ets2-SE^−/−^ mice at E18.5 and P0. The black arrow indicates a VSD. The number in the lower right corner represents the frequency of the deformity. Image scale bars, 1 mm. **c** H&E staining of heart sections at E14.5 showing delayed OFT development in Ets2-SE^−/−^ and Ets2-SE^+/−^ mice. In the WT heart, the PA and AO open into the RV and LV, respectively. In 2/15 of the Ets2-SE^−/−^ embryos, both the PA and AO open into the RV. A patent ventricular septum is observed in 9/15 of Ets2-SE^−/−^ mice and 4/15 of Ets2-SE^+/−^ mice. Image scale bars, 0.5 mm. **d** Compared to those of WT hearts at E12.5, Ets2-SE^−/−^ hearts exhibited smaller RVs. The size of the LV is indicated by a Cambridge blue outline, while the pink outline surrounds the size of the RV. The outline of the PA is indicated by blue lines, while red lines indicate the outline of the AO. WT, *n* = 6; Ets2-SE^+/−^, *n* = 9; Ets2-SE^−/−^, *n* = 7. Low-power image scale bars, 5 mm; high-magnification image scale bars, 1 mm. All error bars are the means ± standard deviations. ***P < 0.001. **e** Volcano plot illustrating differentially expressed genes between Ets2-SE^−/−^ and WT mice at E12.5. Blue, downregulated genes. Red, upregulated genes. **f** Bar plots displaying representative GO terms enriched in downregulated genes in the hearts of E12.5 Ets2-SE^−/−^ mice. **g** Gene set enrichment analysis (GSEA) revealed that the downregulated genes were enriched in biological processes, including homeostasis of the number of cells, and the upregulated genes were enriched in the negative regulation of cell development. **h** Heatmap illustrating scaled expression of genes associated with heart development in WT and Ets2-SE^−/−^ embryonic hearts at E12.5. The color from dark blue to dark red indicates the scaled expression from low to high. FC fold change, ES enrichment score, NES normalized enrichment score, FDR, false discovery rate.

Previous studies have shown that *Ets2* deficiency in mice results in retarded and smaller embryos, abnormal coronary artery development, and thin ventricular walls^44,49^. We examined the cardiac phenotype of Ets2-SE-deleted mice during embryonic development to validate whether Ets2-SE plays a comparable role in heart development. The analysis of birth rates and gross external morphology in Ets2-SE-deleted mice did not reveal consistent effects on embryo morphology (Supplementary Fig. 7) or embryo mortality (Fig. 7a). However, a detailed examination of Ets2-SE^−/−^ and Ets2-SE^+/−^ embryos or neonates at E18.5 and P0 revealed that membranous VSD occurred in one of ten Ets2-SE^−/−^ embryos at E18.5 and one of seven at P0 (Fig. 7b). At E14.5, a critical period of septal closure, malalignment between the artery and the ventricle was also observed in a portion of (2/15) Ets2-SE^−/−^ mice (Fig. 7c). Specifically, both the aorta and pulmonary artery originated from the RV in Ets2-SE^−/−^ embryo hearts, while the pulmonary artery originated from the RV and the aorta originated from the LV in normal hearts (Fig. 7c). However, this severe cardiac malformation did not result in embryonic lethality, possibly because the deletion of Ets2-SE delayed, rather than completely blocked, OFT remodeling at E14.5. This hypothesis also explains the low penetrance in Ets2-SE^−/−^ neonatal mice. Furthermore, we found a significant patent ventricular septum in the majority (9/15) of Ets2-SE^−/−^ embryos at E14.5. At E12.5, we noted that a significantly smaller RV occurred in the Ets2-SE^−/−^ embryos (Fig. 7d). Additionally, a portion (4/15) of the Ets2-SE^+/−^ embryos also exhibited a significantly patent ventricular septum (Fig. 7c). These observations indicate that the deletion of Ets2-SE led to dysplasia of the OFT.

We conducted a transcriptome comparison between Ets2-SE^−/−^ and wild-type mouse embryos at E12.5 using bulk RNA-seq to explore the genes potentially regulated by ETS2 that could contribute to the observed phenotypes. A total of 2290 upregulated and 2204 downregulated genes were detected in the E12.5 hearts of Ets2-SE^−/−^ mice compared to those of wild-type mice (Fig. 7e) (Supplementary Table 7). Downregulation of *Ets2* resulted in the dysregulation of genes involved in heart morphogenesis and cell proliferation (Fig. 7f, g). In addition, ETS2 regulated the expression of critical genes involved in heart development, including *Wnt11* and *Tbx18* (Fig. 7h). WNT11 is essential for second heart field progenitor development and cardiac chamber development^50,51^, while TBX18 plays a role in regulating the development of the epicardium and coronary vessels^52^. In conclusion, these observations reveal that Ets2-SE plays a role in heart development, especially in the OFT, by controlling *Ets2* expression within the heart.

## Discussion

In this study, we revealed a novel and significant role for SMC3 in establishing promoter–enhancer loops to regulate the expression of SE-associated genes crucial for OFT development. We found that a majority of CdLS patients with *SMC3* mutations suffered from CHD, with most exhibiting aorta and pulmonary artery malformations. The formation of the aorta and pulmonary artery results from the correct rotation and separation of the OFT. The various OFT defects observed in SMC3-cKO mice provide a pathological explanation for CHD in CdLS patients. Based on our findings, we speculated that SMC3 might promote the activity of heart-specific SEs to control the expression of master genes essential for OFT development, such as *Notch2*. NOTCH2 is required for proper OFT development^53^. *Jag1* and *Notch2* double heterozygous mice exhibit RV hypoplasia, pulmonary stenosis, VSD, and ASD^54^. This study provides new insight into the mechanism by which the expression of *Notch2* is regulated during OFT development. Furthermore, we highlight the increasing significance of noncoding mutations in individuals with CHD and make a valuable contribution to the recognition of the importance of mutations located in SEs that regulate the expression of master genes in heart development.

During heart development, the fates of various cardiac progenitor cells undergo redefinition to ensure the structural formation and functional stability of the OFT. SEs play a significant role in this process, as they promote robust transcriptional activation of genes within cell fate determination regulatory networks. Our study revealed that several genes, including *ETS2*, *ANKRD1*, and *GADD45G*, are regulated by SMC3 and heart-specific SEs and may be involved in OFT development. ETS2, which is essential for controlling the formation of the anterior–posterior pattern, primitive streak, mesoderm from the ectoderm in the murine embryo^55^ and normal development of the second heart field^56^, synergizes with MESP1 to reprogram human dermal fibroblasts into cardiac progenitors^43^. *Ets2* deficiency leads to abnormal coronary artery development^44^. ANKRD1, which is normally localized to cardiomyocyte sarcomeres, is thought to function as a transcriptional cofactor and negative regulator of myocardial gene expression during heart development. Overexpression of *Ankrd1* in cardiomyocytes leads to abnormal rotation of the early OFT^57^. GADD45G, a member of the GADD45 protein family, is involved in p38 mitogen-activated protein kinase-dependent cell death^58^. Overexpression of *Gadd45g* in mice induces cardiac muscle cell apoptosis and myocardial fibrosis^59^, which contribute to the development of several types of CHD^60,61^. These genes have previously been linked to various aspects of cardiac development and function, and although the specific roles of these genes in OFT development are unclear, our study offers a potential link that can help shed light on their involvement in this crucial process.

*ETS2*, located on chromosome 21, has been extensively studied in various biological processes, including cell proliferation and apoptosis^62,63^. As previously described, *ETS2* is highly expressed in patients with heart failure^64^, and a reduction in *ETS2* expression is associated with improved contractile dysfunction in patients with cardiac hypertrophy^65^. In the present study, we elucidated that high *Ets2* expression in the heart is regulated by Ets2-SE and SMC3, providing potential insights for precision therapy of heart failure patients.

The function of isolated SMC3 mainly depends on cohesin. The dual roles of cohesin in heart development should be considered. While cohesin mediates sister chromosome cohesion, which is essential for cell survival and proliferation, its role in regulating gene expression through chromatin organization is equally important^23^. The phenotype observed in *Smc3*^fl/fl^; *Nkx2.5-Cre* mice may be mainly caused by defects in cohesin function, which leads to cell death after SMC3 deletion. However, the defects in cardiac development experienced by CdLS patients may not result from impaired cell cycling but from abnormal regulation of gene expression because they have a normal *SMC3* allele. During early heart development, the same heart defects in *Smc3*^fl/fl^; *Nkx2.5-*Cre embryos were observed in a small proportion of *Smc3*^fl/+^; *Nkx2.5-*Cre embryos. We hypothesize that SMC3 knockout may affect cell proliferation and lead to dysregulated gene expression, which contributes to the observed heart defects. Differentiating between these two functions of cohesin by phenotypic observation alone may not be possible. CdLS is a genetically heterogeneous pleiotropic disorder with multiple structural and functional deficits, including CHD^22^. A genotype‒phenotype correlation analysis of CdLS revealed that *SMC3* missense mutations and in-frame deletions may lead to a milder phenotype, whereas *NIPBL* truncating pathogenetic mutations result in a more severe phenotype^14^. Our identification of rare and pathogenic *SMC3* variants in individuals with isolated CHD suggests that mild functional variants of genes encoding cohesin members can cause CHD without a classic CdLS phenotype, expanding our understanding of cohesinopathy-related disorders and variants associated with isolated CHD.

In conclusion, our study has shed light on the regulatory role of SMC3 in cardiac development and provides compelling evidence that SMC3 facilitates interactions between promoters and SE loops, thereby regulating the expression of SE-associated genes essential for heart development. These findings contribute to the understanding of the molecular basis of cardiac development and provide valuable insights into the pathogenesis of CHD. Furthermore, our research underscores the elevated risk of CHD in individuals with *SMC3* mutations and strongly supports the inclusion of *SMC3* mutation testing within the panel of genes considered for CHD screening.

## Supplementary information

Supplementary Information

Supplementary Table 3

Supplementary Table 4

Supplementary Table 5

Supplementary Table 6

Supplementary Table 7

## Data Availability

The data that support the findings of this study are available from the corresponding author upon reasonable request. All the raw high-throughput sequence data are available in the GEO (GSE242974). All research materials listed in the Methods section are also included in the major resources table (Supplementary Table 1). The ChIP-seq data were downloaded from the GEO database and are displayed in Supplementary Table 1.
