# Supplementary material for: SMC3 contributes to heart development by regulating super-enhancer associated genes
Source: Exp Mol Med. 2024 Aug 1;56(8):1826–42. doi: 10.1038/s12276-024-01293-0 (PMC11372143; doi:10.1038/s12276-024-01293-0)
Supplement: Supplementary file 1 — Supplementary Information [file 12276_2024_1293_MOESM1_ESM.pdf]

Supplementary information

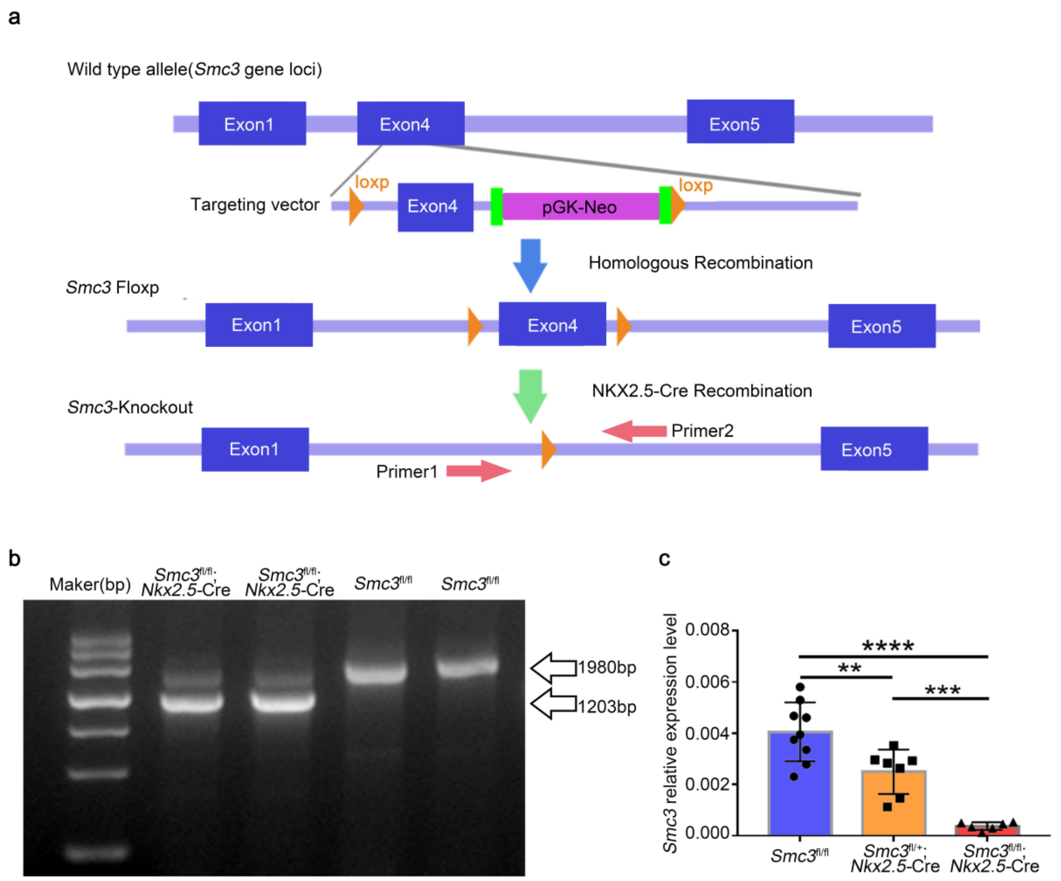

d

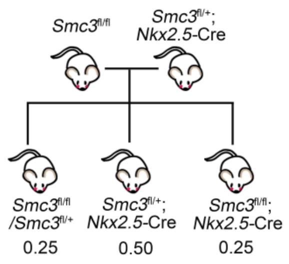

Embryonic / Postnatal day	No.of individuals			Total
	Smc3$^{fl/fl}$/*Smc3*$^{fl/+}$	*Smc3*$^{fl/+}$; *Nkx2.5*-Cre	*Smc3*$^{fl/fl}$; *Nkx2.5*-Cre	
E10.5	14<br>0.33(32%)	20<br>0.47(6%)	9<br>0.21(16%)	43<br>1
E11.5	7<br>0.32(28%)	9<br>0.41(18%)	6<br>0.27(8%)	22<br>1
E12.5	9<br>0.26(35%)	16<br>0.46(8%)	10<br>0.29(16%)	35<br>1
E13.5	9<br>0.35(39%)	14<br>0.54(2%)	3<br>0.12(52%)	26<br>1
E14.5	15<br>0.41(64%)	22<br>0.59(18%)	0<br>0.00(100%)	37<br>1
E15.5	19<br>0.42(69%)	26<br>0.58(16%)	0<br>0.00(100%)	45<br>1
P7	102<br>0.39(56%)	157<br>0.61(22%)	0<br>0.00(100%)	259<br>1

Supplementary Fig. 1. Generation of heart-specific *Smc3*-knockout mice.

a. Generation of heart-specific *Smc3*-knockout mice using the Cre-loxP system as described in the methods.

b. Agarose gel electrophoresis profiles illustrating that heart DNA samples of *Smc3*$^{fl/fl}$;

Nkx2.5-Cre mice at E10.5 are dominated by knockout bands at 1,203 bp in size. A 1,980 bp size single band appears in *Smc3*$^{fl/fl}$ embryonic heart DNA samples. The primers used for PCR are primer 1 and primer 2 as shown in A.

c. The qPCR results from *Smc3* mRNA extracted from embryonic heart of *Smc3*$^{fl/fl}$ and *Smc3*$^{fl/fl}$*; Nkx2.5*-Cre at E10.5.

d. Genotype distribution of individuals resulting from *Smc3*$^{fl/fl}$ and *Smc3*$^{fl/+}$; *Nkx2.5*-Cre crossing. The percentage of *Smc3*$^{fl/fl}$; *Nkx2.5*-Cre was significantly reduced at E13.5 (12% vs. 25%), which was not applicable to Mendel's law. The numbers in parentheses represent the deviation of the actual ratio from the theoretical ratio in mice representing each genotype.

Error bars indicate mean ± standard deviation. $^{**}p < 0.01$, $^{***}p < 0.001$ and $^{****}p < 0.0001$.

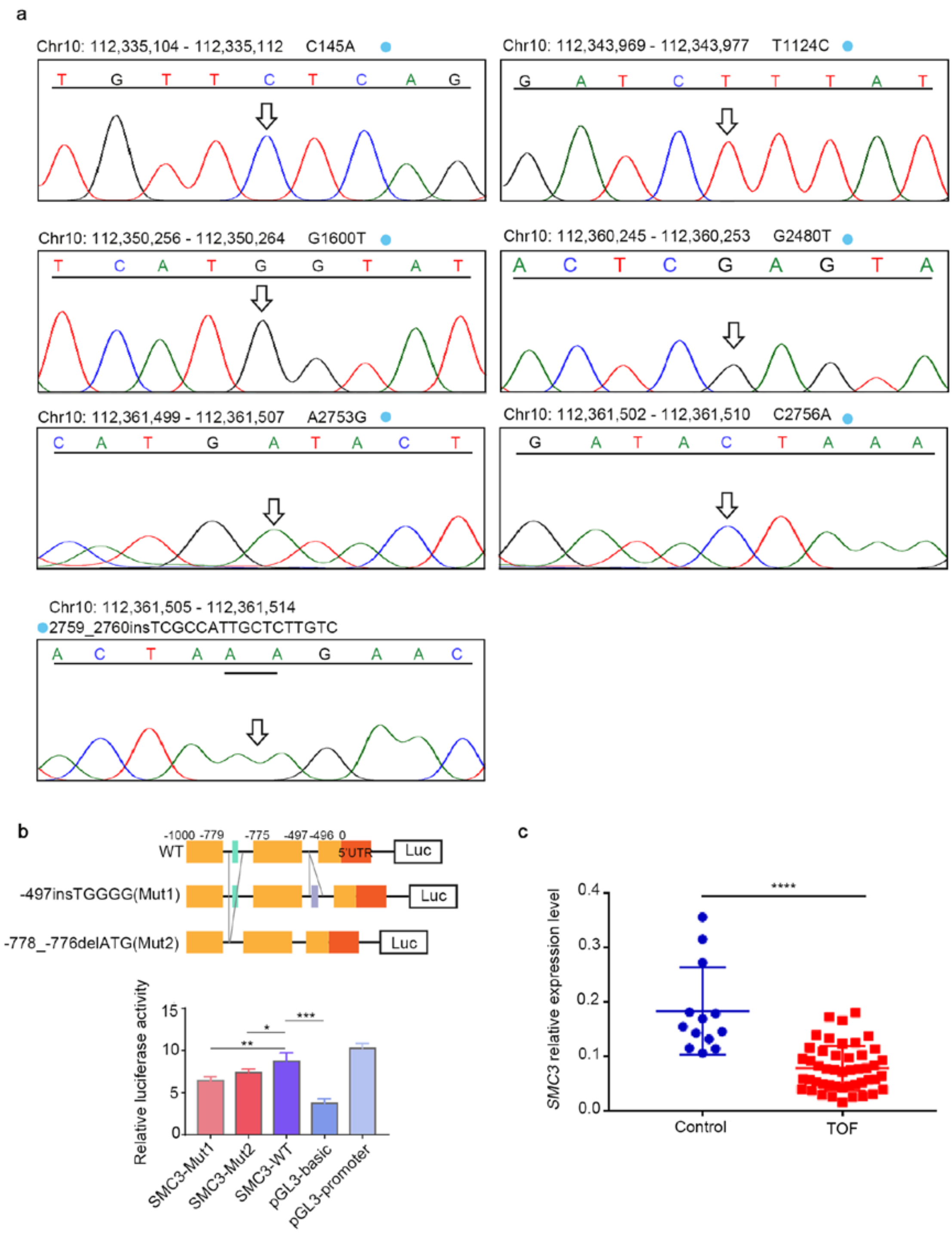

Supplementary Fig. 2. Sequence and functional verification of *SMC3* variants

a. Normal sequence peak profiles including the identified SMC3 coding variant sites within 104 Han Chinese controls. The seven SMC3 coding variants of patients with isolated CHD from our peers' WES data were not detected in 104 Han Chinese controls. The black arrow points to the site of the variant.

b. Validation of the effect of variants in the regulatory regions on *SMC3* gene expression. The schema of *SMC3* promoter region for constructing luciferase reporter plasmids (Top). The orange regions indicate the 5'UTR of *SMC3*. The light yellow regions indicate *SMC3* promoter regions. The purple and green rectangles indicate the location of two *SMC3* variants (-498insTGGGG and -778_-776delATG), respectively. Dual luciferase reporter assay revealed that promoter activity markedly reduced when *SMC3* pomoter had the variants.

c. *SMC3* was downregulated in heart tissues from patients with TOF (n=47) compared with controls (n=13) by qPCR assay.

All error bars are mean ± standard deviation. *P < 0.05, **P < 0.01, ***P < 0.001, and ****P < 0.0001. SMC3-Mut1, the pGL3-basic plasmid including *SMC3* promoter region with -498insTGGGG variant; SMC3-Mut2, the pGL3-basic plasmid including *SMC3* promoter region with -778_-776delATG variant; SMC3-WT, the pGL3-basic plasmid including wild type *SMC3* promoter region.

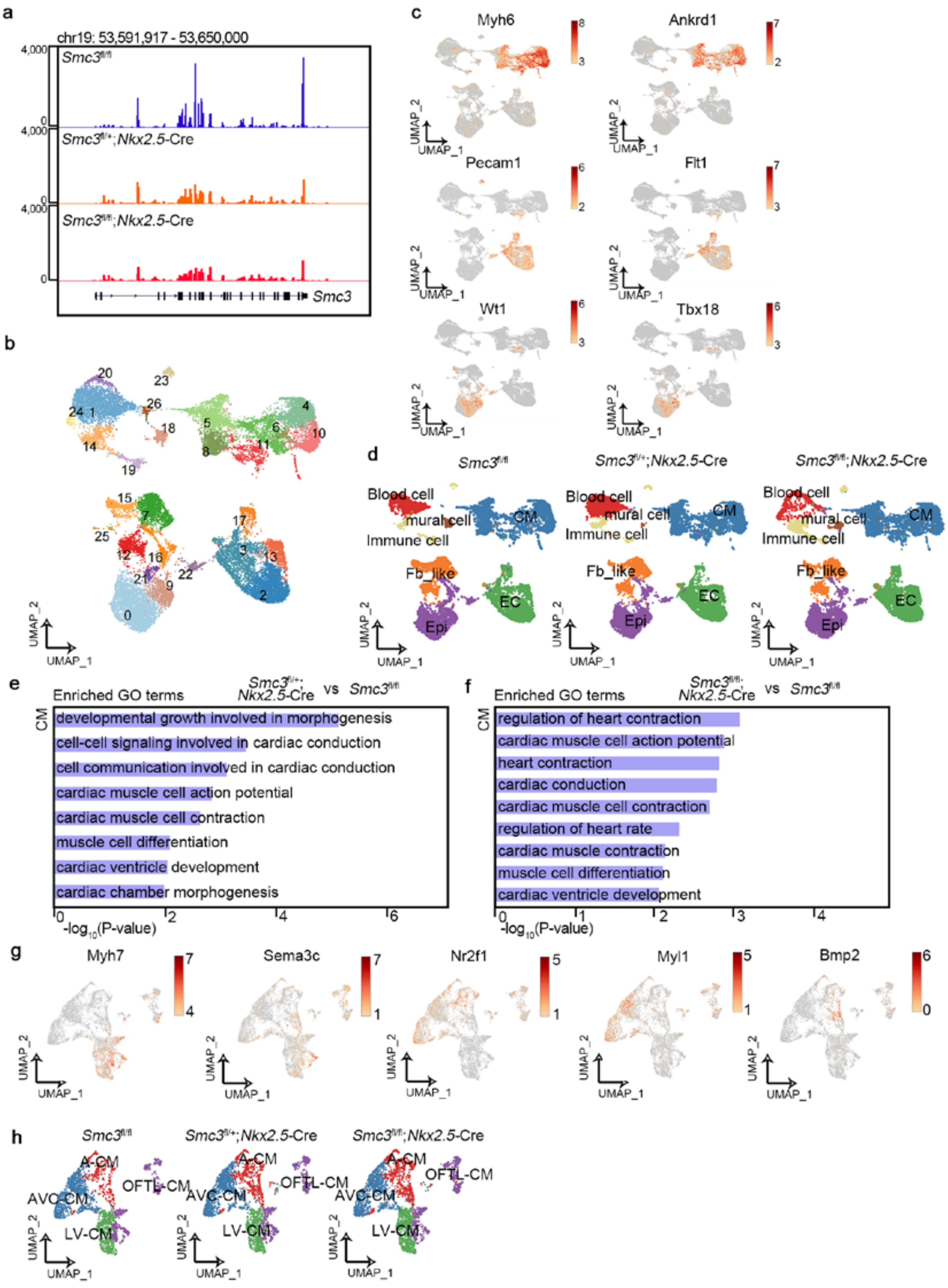

Supplementary Fig. 3. The partial analysis on the data of snRNA-seq.

a. Visualization of snRNA-seq at *Smc3* loci validated the *Smc3* knockout efficiency of *Smc3*-cKO embryos cardiac tissue using integrative genomics viewer.

b. Unsupervised clustering of snRNA-seq datasets from E12.5 *Smc3*$^{fl/fl}$, *Smc3*$^{fl/+}$; *Nkx2.5*-Cre, and *Smc3*$^{fl/fl}$; *Nkx2.5-Cre* embryos cardiac tissue.

c. UMAP feature plots for marker genes of CM, Epi, and EC, respectively. The color from dark to light represents the average expression from high to low.

d. UMAP feature plot of cardiac tissue single cell transcriptomes in *Smc3*$^{fl/fl}$, *Smc3*$^{fl/+}$; *Nkx2.5*-Cre, and *Smc3*$^{fl/fl}$; *Nkx2.5-Cre* embryos.

e. and f. Bar plots displaying representative GO terms enriched in downregulated genes in CM from E12.5 *Smc3*$^{fl/+}$; *Nkx2.5*-Cre (E) and *Smc3*$^{fl/fl}$; *Nkx2.5-Cre* (F) cardiac tissue.

g. UMAP feature plots for marker genes of CM subtypes. The color from dark to light represents the average expression from high to low.

h. UMAP feature plot of CM single cell transcriptomes in *Smc3*$^{fl/fl}$, *Smc3*$^{fl/+}$; *Nkx2.5*-Cre, and *Smc3*$^{fl/fl}$; *Nkx2.5-Cre* embryos cardiac tissue.

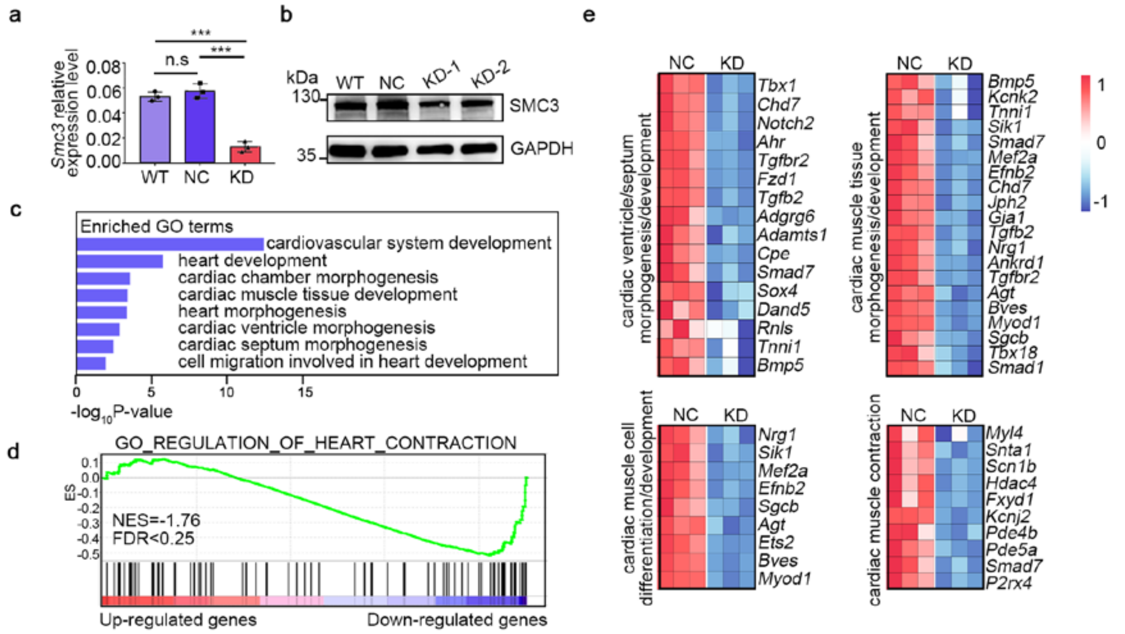

Supplementary Fig. 4. SMC3 regulates gene expression in mouse cardiomyocyte cell line.

a and b. The qPCR (a) and WB (b) analysis validated the *Smc3* knockdown efficiency oin HL-1 cells.

c. GO analysis showing downregulated genes in KD are enriched in pathways involved in cardiac development.

d. GSEA illustrating downregulated genes in KD enriched to the biology process of regulation of heart contraction.

e. Heatmap showing the scaled expression of genes associated with heart development in NC and KD. The color from dark blue to dark red indicates the scaled expression from low to high.

All error bars are mean ± standard deviation. ***P < 0.001.

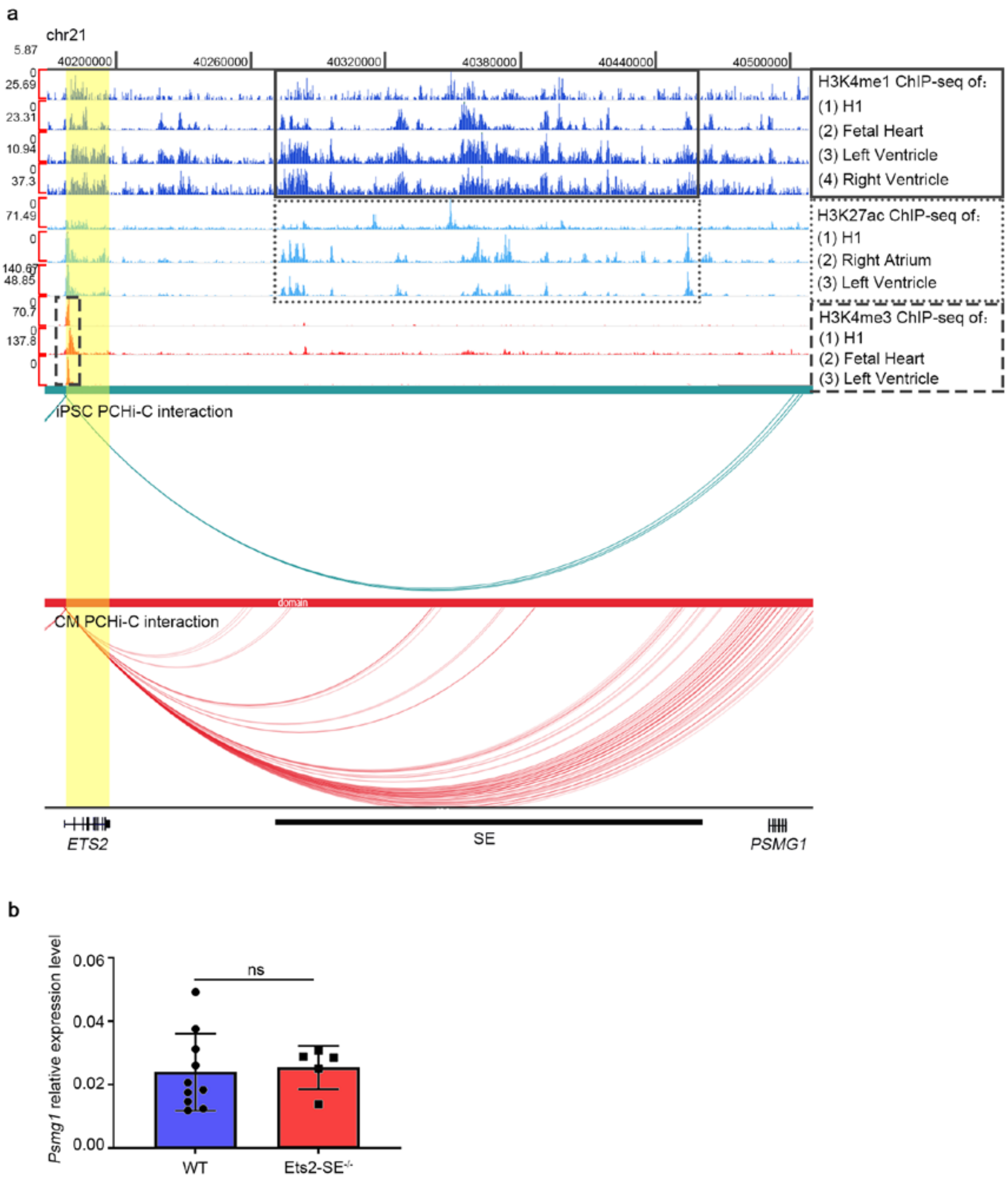

Supplementary Fig. 5. Super-enhancer regulates *Ets2* expression in the heart.

a. Normalized ChIP-seq profiles of H3K4me1, H3K27ac, and H3K4me3 from top to bottom, respectively. TAD interactions in induced pluripotent stem cells (iPSC) (Green) and CM (Red) as detected by promoter capture Hi-C (PCHi-C). The yellow area represents the *ETS2* locus. Sequences with high occupancy in H3K4me1 and H3K27ac represent the position of the SE. Sequences with high occupancy in H3K4me3 represent

the position of the promoter.

b. The qPCR results of *Psmg1* mRNA extracted from mouse embryonic heart of WT and Ets2-SE$^{-/-}$ at E10.5. All error bars are mean ± standard deviation.

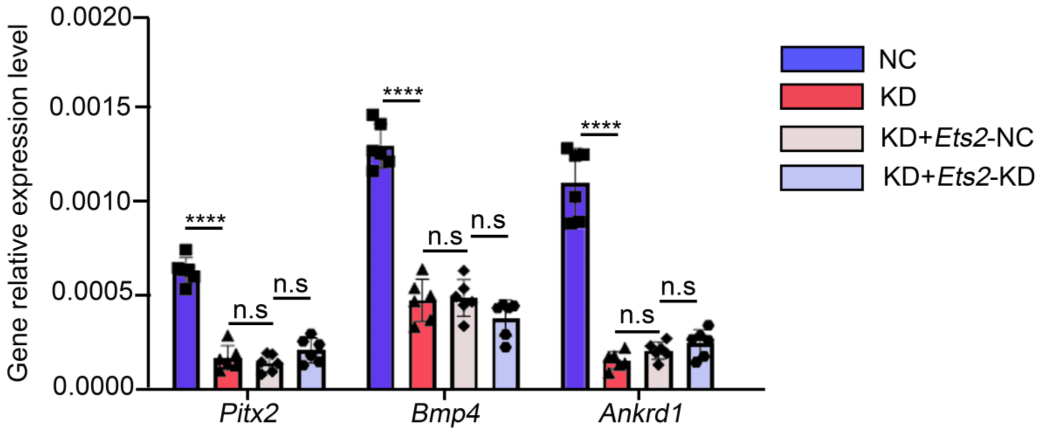

Supplementary Fig. 6. Expression of the genes associated with heart development in four groups of HL-1 cells.

The qPCR analysis of the impact of *Ets2* overexpression on genes in Fig.3f. Compared to the eight genes in Fig.6i, *Ets2* overexpression had no effect on the expression of these three genes. Error bars indicate mean ± standard deviation. ****P < 0.0001.

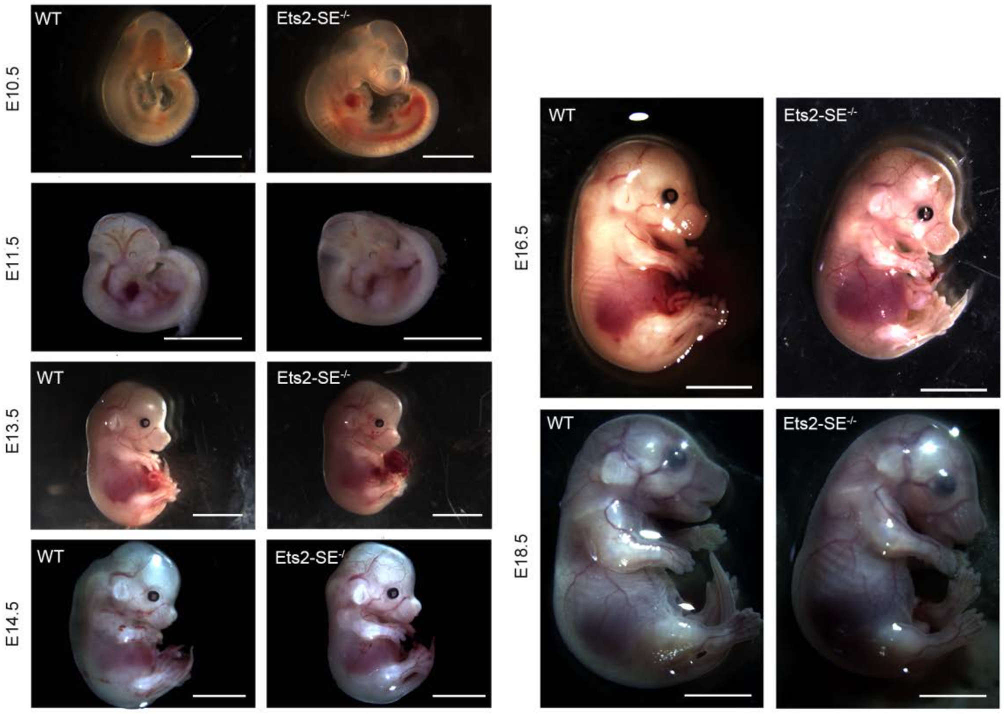

Supplementary Fig. 7. Gross external morphology of wild type and Ets2-SE$^{-/-}$ embryos during the mouse heart development.

Analysis of gross external morphology at different developmental times revealed that Ets2-SE-deletion produced no significant effects on embryo morphology. Image at E10.5 scale bars, 2mm; Image scale bars at E11.5, E13.5, E14.5, E16.5, and E18.5, 5mm.

Supplementary Table 1. List of primers sequences, antibodies, and ChIP-seq data from GEO database.

Primers for qPCR		
Gene name	Forward (5'-3')	Reverse (5'-3')
human *SMC3*	CAGCAAACTGAGGTACAATCCCA	CCGATGGCTGACTTGGTCAC
mouse *Smc3*	TCACTGCTGGTAACAGGTTATT	CACCTCTCCAGGAAGATTCATT
human *GAPDH*	GGGAAACTGTGGCGTGAT	GAGTGGGTGTCGCTGTTGA
mouse *Gapdh*	CGGGGTCCCAGCTTAGGTTC	CCCAATACGGCCAAATCCGTT
human *ETS2*	AGATGAACTTGCCACTGGAG	CTTGAGTGTCCCTCTGTAACTG
mouse *Ets2*	CCTTCGATGGCTCTCTGTTT	CGAGTCATGGGAGACAGAAT
mouse *Psmg1*	GATGAAGTTGGACCGTGTCA	CTGTGCTTTCAGGAATGTTCTTC
mouse *Myl1*	CCCAGCAATGAAGAGATGAATG	TGGTCCTTGTTGTTGGAGATAG
mouse *Myl4*	AACCCAAGCCTGAAGAGATG	TCCACGAAGTCCTCATAGGT
mouse *Myl9*	CAGAACCGAGATGGCTTCATT	GTTCATCATGCCCTCCAGATAC
mouse *Hand2*	CTGGCCACCAGCTACATC	TTCAGCTCTTTCTTCCTCTTCTC
mouse *Vangl2*	GATGAGCGGGATGACAACT	GTGAGGTCATCATGGGAGATAC
mouse *Tnnt2*	GATGCTGAAGAAGGTCCAGTAG	GTCATCAAAGTCCACTCTCTCTC
mouse *Tnnc1*	CTACAAAGCTGCGGTAGAACA	CGCCCAGGACAAAGATATCAA
mouse *Actc1*	TGTTCCCATCTATGAGGGTTATG	GCAGTGGTGACAAAGGAGTA
mouse *Pitx2*	CCTGAAGTCGCAGAGAAAGATAA	GGCTAGTGAAATGAGTCCTCTG

mouse *Bmp4*	TCCATCACGAAGAACATCTGG	GATCACCTCATTCTCTGGGATG
mouse *Ankrd1*	CATGGACATGCTAGTGCTAGAG	GCTCTTGGCCCTTAACCTT
Primers for mouse genotyping identification		
Cre	CAGAACCTGAAGATGTTCGCGAT	CCTGATCCTGGCAATTTCGGC
loxP	CACCCAGGCCTTTCTTAGTTCC	TATCTGGCCCTTATGTAGTA
loxP for verifying	GGAAACCAGCGCTTAGTAGGA	GACAGAGACGCAGGGAAGCAAATG
Ets2-SE-F1	CCACGGCCACTCTTATTCTC	TGTGCCTCTGCAGCATGAAC
Ets2-SE-F2	ACAGTGGGTCCCTAGAAGTG	GGCCTTTAAGACCTTCATCC
Primers for ChIP-qPCR		
Ets2-promoter	ATGCACTGACCCTGAAGATG	TCCGGGAATGTTTCTGATGG
Primers for 3C-HTGTS		
Mouse-*Ets2* - MboI Biotin-Primer	GGACCTGCAGACAGCCTAAC	
Mouse-*Ets2* - MboI nested Primer	CTGGAGTTCAGACGTGTGCTCTTCCGATCTAGCTGCAGGGTCGCAGAGAA	
HTGTS adapter UP	GCGACTATAGGGCACGCGTGGNNNNNN-NH2	

HTGTS adapter down	/5-Phosphorylation/CCACGCGTGCCCTATAGTCGC-NH2		
Antibodies			
	Company	Reference	Dilution
Anti-SMC3	Abcam	ab9263	For ChIP assay: 2μL /10.0×10^{6} cells, For WB: 1:1000
Anti-ETS2	Abcam	ab272866	For ChIP assay: 2μL /10.0×10^{6} cells, For WB: 1:1000
Anti-GAPDH	Proteintech	60004-1-Ig	For WB: 1:2000
Anti -H3K4me3	Millipore	07-473	For ChIP assay: 1μL / 10.0×10^{6} cells
Anti-H3K27ac	Millipore	07-449	For ChIP assay: 1μL / 10.0×10^{6} cells
Anti-NOTCH2	CST	D76A6	For WB: 1:1000
Anti-ANKRD1	Proteintech	11427-1-AP	For WB: 1:1000
Anti-GADD45G	Affinity Biosciences	DF2376	For WB: 1:1000
Anti-β-Tubulin	Proteintech	10094-1-AP	For WB: 1:2000
ChIP-seq data from GEO database			
Protein	Species	Biological Sources	GEO

			accession
H3K4me1	Homo sapiens	ESC H1 cells	GSM409307
H3K4me1	Homo sapiens	fetal heart	GSM706848
H3K4me1	Homo sapiens	left ventricle	GSM906404
H3K4me1	Homo sapiens	right ventricle	GSM1059445
H3K27ac	Homo sapiens	ESC H1 cells	GSM663427
H3K27ac	Homo sapiens	Right Atrium	GSM906396
H3K27ac	Homo sapiens	left ventricle	GSM1013124
H3K4me3	Homo sapiens	ESC H1 cells	GSM432392
H3K4me3	Homo sapiens	fetal heart	GSM772735
H3K4me3	Homo sapiens	left ventricle	GSM906406
SMC3	Mus musculus	ESC cells	GSM2111724
RAD21	Mus musculus	ESC cells	GSM591469
CTCF	Mus musculus	embryonic heart	GSM851286
H3K4me3	Mus musculus	embryonic heart	GSM1944082
H3K27ac	Mus musculus	heart ventricle	GSM2577068
H3K27ac	Mus musculus	embryonic heart	GSM851290

Supplementary Table 2. Frequency of CHD in CdLS cases with pathogenic gene mutation.

Pathogenic gene	Frequency (CdLS cases with CHD / CdLS cases)	Type of CHD
SMC3	52.38% (22/42) [1-6]	PDA, VSD, PPS, TOF and so on.
NIBPL	26.73% (58/217) [2,3,7,8]	PS+ASD, TOF, VSD and so on.
RAD21	25.00% (11/44) [2,3,7,9-11]	ASD, PDA, PFO, TOF, subaortic stenosis and so on.
SMC1A	24.14% (14/58) [2,3,12-16]	PS+ASD, ASD, heart murmur, PS and so on.
HDAC8	16.33% (8/49) [2,5,8,17-20]	PS, atrial septum aneurysm and so on.

Abbreviation: CHD: congenital heart disease; CdLS: Cornelia de Lange syndrome; PDA: patent ductus arteriosus; ASD: atrial septal defect; PS: pulmonry stenosis; AS: aortic stenosis; BAV: bicuspid aortic valve; PPS: Peripheral pulmonary stenosis; TOF: tetralogy of Fallot; PFO: patent foramen ovale.

References

1 Gil-Rodríguez, M. C. *et al.* De novo heterozygous mutations in SMC3 cause a range of Cornelia de Lange syndrome-overlapping phenotypes. *Hum Mutat* **36**, 454-462 (2015).

2 Mannini, L., Cucco, F., Quarantotti, V., Krantz, I. D. & Musio, A. Mutation spectrum and genotype-phenotype correlation in Cornelia de Lange syndrome. *Hum Mutat* **34**, 1589-1596 (2013).

3 Ansari, M. *et al.* Genetic heterogeneity in Cornelia de Lange syndrome (CdLS) and CdLS-like phenotypes with observed and predicted levels of mosaicism. *J Med Genet* **51**, 659-668 (2014).

4 Yuan, B. *et al.* Global transcriptional disturbances underlie Cornelia de Lange syndrome and related phenotypes. *J Clin Invest* **125**, 636-651 (2015).

5 Liu, C. *et al.* Analysis of clinical and genetic characteristics in 10 Chinese individuals with Cornelia de Lange syndrome and literature review. *Mol Genet Genomic Med* **8**, e1471 (2020).

6 Li, R. *et al.* A Chinese Case of Cornelia de Lange Syndrome Caused by a Pathogenic Variant in SMC3 and a Literature Review. *Front Endocrinol (Lausanne)* **12**, 604500 (2021).

7 Pié, J. *et al.* Special cases in Cornelia de Lange syndrome: The Spanish experience. *Am J Med Genet C Semin Med Genet* **172**, 198-205 (2016).

8 Li, Q. *et al.* Clinical and molecular analysis in a cohort of Chinese children with Cornelia de Lange syndrome. *Sci Rep* **10**, 21224 (2020).

9 Deardorff, M. A. *et al.* RAD21 mutations cause a human cohesinopathy. *Am J*

Hum Genet **90**, 1014-1027 (2012).

10 Krab, L. C. *et al.* Delineation of phenotypes and genotypes related to cohesin structural protein RAD21. *Hum Genet* **139** 575-592, (2020).

11 Dorval, S. *et al.* A novel RAD21 mutation in a boy with mild Cornelia de Lange presentation: Further delineation of the phenotype. *Eur J Med Genet* **63**, 103620 (2020).

12 Yuan, B. *et al.* Clinical exome sequencing reveals locus heterogeneity and phenotypic variability of cohesinopathies. *Genet Med* **21**, 663-675 (2019).

13 Wenger, T. L. *et al.* Novel findings of left ventricular non-compaction cardiomyopathy, microform cleft lip and poor vision in patient with SMC1A-associated Cornelia de Lange syndrome. *Am J Med Genet A* **173**, 414-420 (2017).

14 Deardorff, M. A. *et al.* Mutations in cohesin complex members SMC3 and SMC1A cause a mild variant of cornelia de Lange syndrome with predominant mental retardation. *Am J Hum Genet* **80**, 485-494 (2007).

15 Gervasini, C. *et al.* Cornelia de Lange individuals with new and recurrent SMC1A mutations enhance delineation of mutation repertoire and phenotypic spectrum. *Am J Med Genet A* **161a**, 2909-2919 (2013).

16 Limongelli, G. *et al.* Hypertrophic cardiomyopathy in a girl with Cornelia de Lange syndrome due to mutation in SMC1A. *Am J Med Genet A* **152a**, 2127-2129 (2010).

17 Mio, C. *et al.* A novel de novo HDAC8 missense mutation causing Cornelia de Lange syndrome. *Mol Genet Genomic Med* **9**, e1612 (2021).

18 Meshram, G. G., Kaur, N. & Hura, K. S. Cornelia De Lange Syndrome In A 4-Year-Old Child From India: Phenotype Description And Role Of Genetic Counseling. *Med Arch* **72**, 297-299 (2018).

19 Deardorff, M. A. *et al.* HDAC8 mutations in Cornelia de Lange syndrome affect the cohesin acetylation cycle. *Nature* **489**, 313-317 (2012).

20 Kaiser, F. J. *et al.* Loss-of-function HDAC8 mutations cause a phenotypic spectrum of Cornelia de Lange syndrome-like features, ocular hypertelorism, large fontanelle and X-linked inheritance. *Hum Mol Genet* **23**, 2888-2900 (2014).
